# Conservative evolution of genetic and genomic features in *Caenorhabditis becei*, an experimentally tractable gonochoristic worm

**DOI:** 10.1101/2025.05.09.653148

**Published:** 2025-05-15

**Authors:** Jose Salome Correa, Luke M. Noble, Solomon A. Sloat, Tuc H. M. Nguyen, Matthew V. Rockman

**Affiliations:** 1. Department of Biology and Center for Genomics & Systems Biology, New York University, New York, New York; 2. EnviroDNA, Melbourne, Australia; 3. Department of Biology, University of North Carolina, Chapel Hill, North Carolina

**Keywords:** *Caenorhabditis*, genome evolution, GC content, *Medea* elements, genetic maps

## Abstract

*Caenorhabditis* nematodes represent a promising model clade for evolutionary genetics and genomics, but research has focused on the three androdioecious species, those with self-fertile hermaphrodites, all in the Elegans Group of species. The majority of *Caenorhabditis* species are gonochorists, with males and females, characterized by inconveniently high heterozygosity and inbreeding depression. We have identified *C. becei*, a Japonica Group species from Panamá, as an experimentally tractable gonochorist. We describe a new chromosomal genome assembly of a healthy inbred *C. becei* reference strain, integrating data from PacBio HiFi reads, Illumina short reads, genetic linkage, and HiC chromatin contacts, and experimental gene annotation with short- and long-read data. Several genetic properties that are well characterized in the Elegans Group are present in this Japonica Group species: the organization of the genetic map, cosegregation of autosomal indels and sex chromosomes, and segregation distortion due to *Medea* elements, demonstrated here for the first time in a gonochoristic *Caenorhabditis* species. Some aspects of the genome are highly conserved, including synteny across the six chromosomes and the distributions of repetitive sequences and genes along each chromosome. Other features are quite distinctive, including evolved shifts in GC composition & heterogeneity along the genome. Both codon & amino acid usage are shifted in concert with the species’ genomic GC content. *C. becei* has an unusually large X chromosome, which we find is associated with multiple local gene family expansions. These findings and resources lay the foundation for further experimental and computational studies of *Caenorhabditis* genetics and genomics.

## INTRODUCTION

*Caenorhabditis elegans* is among our most powerful experimental model organisms, and decades of intensive study have revealed innumerable details about its cellular, molecular, and developmental biology. *C. elegans* sits within a speciose clade of morphologically similar nematodes that share many features, down to the number and arrangement of cells (Memar et al., 2019; Zhao et al., 2008). Some pairs of reproductively isolated species completely lack distinguishing morphological features (Sudhaus & Kiontke, 2007). Most *Caenorhabditis* species share ecological attributes as well, reproducing within patches of decomposing plant matter and feeding on bacteria. Despite the morphological and ecological conservatism of *Caenorhabditis* nematodes, they are extremely divergent at the sequence level; distantly related *Caenorhabditis* are more diverged at the level of protein sequences than the most distantly related vertebrates or insects (Kiontke et al., 2004).

The combination of conservation and divergence makes *Caenorhabditis* a particularly attractive model for comparative genomics and genetics, as factors that can confound evolutionary analysis in other groups – life history, trophic level, body size, and so forth – hardly vary within this clade (Kiontke et al., 2011; Stevens et al., 2019). Moreover, comparative experimental analysis of many species under identical conditions is straightforward, as more than 70 species are available in culture and compatible with standard laboratory growth conditions (Daul et al., 2019).

Despite these advantages, comparative experimental and population genetics has focused largely on a handful of species closely related to *C. elegans*, within the Elegans Group in the phylogeny. Further, the bulk of research has focused on the three species, all within the Elegans Group, that exhibit androdioecy, a mating system of self-fertile hermaphrodites and cross-fertile males.

To understand which genetic and genomic features of the *Caenorhabditis* model species are shared more broadly across the clade, we generated a contiguous genome assembly for *C. becei*, an experimentally advantageous species in the Japonica group, sister to the well-studied Elegans group (Stevens et al., 2019). We then tested for conservation, convergence, or divergence in a range of genetic and genomic characteristics.

*C. becei* has many useful properties as an experimental representative of both the Japonica Group and gonochoristic (male/female) *Caenorhabditis* species more broadly. It shares with *C. elegans* (and most *Caenorhabditis*) a compact genome, free of repetitive centromeres or Y-chromosomes, and it is amenable to cryopreservation. It grows well in laboratory culture and so has already proved useful for experimental studies of viral susceptibility, sex ratio, and reproductive biology (Huang et al., 2023; Lamelza et al., 2019; Shaw & Kennedy, 2022). Its genes and genome can be manipulated by microinjection of plasmids or nucleoprotein particles, and it has a rapid generation time, cycling from egg to egg in less than 48 hours at its native temperature of 25°C. *C. becei* is named for its type locality, Barro Colorado Island (BCI), Panamá (Stevens et al., 2019). It is a common species in the rainforest of BCI, the most intensively studied tropical forest on earth, and consequently it holds great promise for ecological studies (Sloat et al., 2022).

*C. becei*’s greatest virtue, however, and the one that motivated its selection as our model species, is that its populations harbor a relatively modest load of deleterious recessive variants, facilitating construction of healthy and fecund inbred lines (preprint forthcoming). For short-lived small-bodied organisms like nematodes, inbred lines are a critical tool for genetic analysis, allowing experimental replication of genotypes – including outbred genotypes generated by crossing inbred lines (Andersen & Rockman, 2022). *C. becei* provides a route to genomic analysis of inbreeding depression, a phenomenon absent from the self-fertile species like *C. elegans* and nearly intractable in hyperdiverse Elegans Group species like *C. brenneri* and *C. remanei* (Adams et al., 2022; Baer et al., 2010; Barrière & Félix, 2005; Dolgin et al., 2007), and to quantitative genetic analysis of outcrossing-related traits, including reproductive behaviors that are vestigial in *C. elegans*.

Previously, we reported a short-read draft assembly of *C. becei* inbred line QG2083 in 1,567 scaffolds (Stevens et al., 2019), and we subsequently used linkage data to consolidate the reference into a 256-scaffold assembly (Lamelza et al., 2019). These fragmented assemblies have already proved valuable in comparative studies of gene and regulatory network evolution (Carelli et al., 2022; Eurmsirilerd & Maduro, 2020; Kursel et al., 2021; Maduro, 2020; Nelson & Ambros, 2021; Fusca et al. 2024). Here, we generate a new assembly, with chromosomal scaffolds, for an independent inbred line, QG2082. We integrated data from PacBio HiFi reads, Illumina short reads, genetic linkage, and HiC chromatin contacts. We describe the assembly of the new *C. becei* reference genome and our experimental and computational annotation of genes and repetitive elements. Taking advantage of *C. becei*’s compatibility with laboratory conditions, we also show that several genetic properties that are well characterized in the Elegans Group are conserved with this Japonica Group species: the organization of the genetic map, segregation distortion due to *Medea* elements, and the pattern of cosegregation of autosomal variants and sex chromosomes.

## RESULTS

### *C. becei* is part of a Neotropical clade of species with narrow ranges

**Figure 1.**
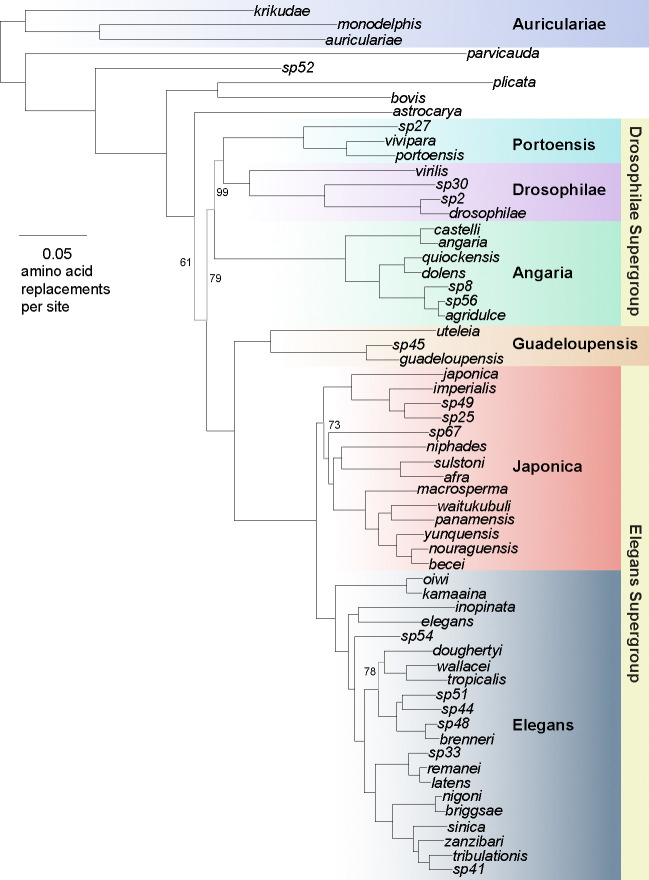
Phylogeny of *Caenorhabditis* inferred from protein sequences of 2,059 single-copy orthologs. Gene trees were estimated by maximum likelihood and the species tree estimated under the multispecies coalescent. The unrooted phylogeny is plotted with the root on the branch connecting the Auriculariae Group to the rest of the species (Dayi et al., 2021; Sloat et al., 2022; Slos et al., 2017; Stevens et al., 2019). Branch lengths are maximum likelihood estimates from the concatenated protein sequences. Named species Groups and Supergroups are indicated. Branch support (quadripartition frequency) is 100% for all branches except where indicated.

To situate *C. becei* within *Caenorhabditis*, we used data from 2,059 single-copy orthologs present in at least 80% of 60 *Caenorhabditis* species to generate the most comprehensive phylogeny of the genus to date (Figure 1). The major outline of the phylogeny, inferred from individual gene trees under the multispecies coalescent model, is largely congruent with previously inferences (Dayi et al., 2021; Kiontke et al., 2011; Sloat et al., 2022; Stevens et al., 2019, 2020), with strong support for the Elegans and Japonica Groups and their union in the Elegans Supergroup. Support for the other named groups is also strong, though the branches connecting them to one another are more equivocal.

*C. becei* falls within a well-supported section of the Japonica Group. The phylogeny shows that *C. becei* evolved within a clade of species that are endemic to narrow geographic ranges in the neotropics. Each species is known from only a single region (*becei* and *panamensis* from Barro Colorado Island, *nouraguensis* and *macrosperma* from French Guiana, *yunquensis* from Puerto Rico, and *waitukubuli* from Dominica), though they are often very abundant locally (Félix et al., 2014; Kiontke et al., 2011; Stevens et al., 2019; Sloat et al., 2022). For example, *C. nouraguensis* is the single most common species observed in French Guiana (Félix et al., 2013; Ferrari et al., 2017). The depth of sampling in French Guiana and BCI is sufficient to conclude that the species found at each locale are likely absent from the other (Sloat et al., 2022). The neotropics are not well sampled otherwise, and many additional species in this clade remain to be discovered.

### The genome of *C. becei*

We assembled a chromosomally scaffolded reference genome for *C. becei* strain QG2082 (Table S1). This inbred line was derived by 25 generations of sib-mating from wild-caught isofemale line QG704, isolated in 2012 from a *Gustavia superba* flower rotting on the forest floor on Barro Colorado Island, and then cryopreserved (Sloat et al., 2022). Assembly integrated PacBio HiFi long reads, Illumina short reads, linkage data from a genetic cross, and HiC chromatin contact data.

The nuclear genome assembly spans 93.9 Mb. DNA sequence statistics, such as GC content, repeat content, and gene density, show symmetrical patterns along the chromosomes and align with recombination rate domains, consistent with good chromosomal completeness and an absence of large-scale assembly errors. Most chromosomes terminate at both ends in oriented telomeric repeat sequences (Figure S1). Chromosomes were assigned identities based on conserved synteny with *C. elegans*.

The mitochondrial genome of *C. becei* is a 13,708 bp circular molecule encoding 12 protein-coding genes, 2 ribosomal RNAs, and 22 transfer RNAs. The genome shares many features with *C. elegans*, with identical gene order and minimal intergenic space.

**Figure 2.**
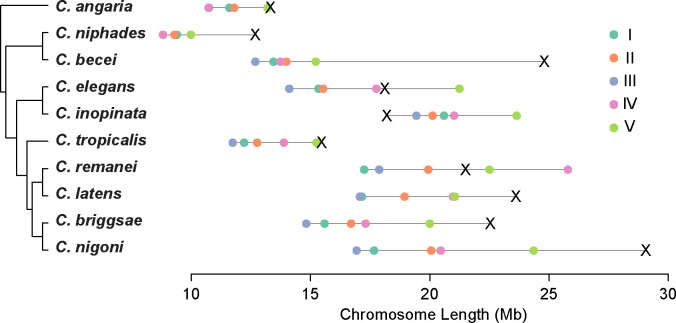
*C. becei* has a small genome but a giant X chromosome. Chromosome sizes are plotted for species of *Caenorhabditis* with published chromosomally scaffolded genomes.

Two characteristics of the *C. becei* genome stand out. First, one chromosome, identified by coverage and synteny as the X chromosome, is much larger than the other chromosomes, almost twice the length of the shortest chromosome and 40% longer than the *C. elegans* X (Figure 2). Second, the GC content of the genome is unusually high and exceptionally heterogeneous.

### Architecture of the genetic map

**Figure 3.**
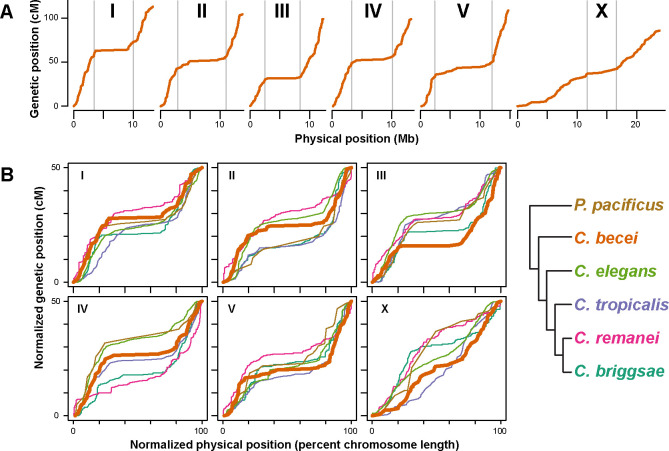
**A.** *C. becei* Marey maps showing effective genetic position (crossovers per chromosome in the G_4_BC_2_ mapping cross) as a function of physical position. Gray bars indicate the arm-center boundaries as estimated by segmented linear regression. **B.** Normalized Marey maps for the six Rhabditid species with complete physical and genetic maps. The phylogeny at right is not to scale; *Pristionchus pacificus* is very distantly related to *Caenorhabditis*.

We characterized the relationship between the genetic map and physical genome, using data from an advanced intercross between inbred lines QG2082 and QG2083 (Lamelza et al., 2019). These inbred lines were derived from independent isofemale lines collected from localities separated by 3 kilometers. The resulting *C. becei* recombination landscape, the first for a *Caenorhabditis* species outside the Elegans group, closely matches the patterns known from *C. elegans*. Each chromosome has a low-recombination central domain flanked by high recombination arms, and in most cases, small tip regions with little or no recombination (Figure 3A, Table S2). The no-recombination tips are very small in *C. becei*, and potentially absent in some cases (or unassembled, although the presence of long telomeric repeats on most chromosomes argues against this).

The overall pattern of recombination rate variation along the autosomes is largely conserved across the *Caenorhabditis* species with characterized recombination maps (Figure 3B). *C. elegans, C. briggsae,* and *C. tropicalis* show concordant domains of zero, high, and low recombination in tips, arms, and centers respectively (Barnes et al., 1995; Noble et al., 2021; Rockman & Kruglyak, 2009; Ross et al., 2011; Stevens et al., 2022); *C. remanei* is exceptional; it shows the typical arm-center organization but has high recombination in the tips (Teterina et al., 2023). Distantly related *Pristionchus pacificus* also shows *Caenorhabditis*-like domain architecture, despite substantial differences in meiotic machinery (Rillo-Bohn et al., 2021; Yoshida et al., 2023).

*C. becei* resembles *C. elegans* and *C. briggsae* in having a distinct if subtle domain structure on the X chromosome, while other species have idiosyncratic or inverted X chromosome domain structure (though the *P. pacificus* X chromosome map may be distorted by inversions: Rillo-Bohn et al., 2021).

We used segmented linear regression to estimate boundaries between the center and arm domains and to estimate recombination rates for each species (Table S3, Figure S2), under the assumption of complete crossover interference (i.e., 50cM chromosomes, as in *C. elegans*). *C. becei* autosome arms have relatively high recombination rates (mean 8.1 cM/Mb, range 6.5–10.9), similar to those of *C. tropicalis* (mean 8.2 cM/Mb) and *C. briggsae* (mean 7.8), slightly higher than those of *C. elegans* (mean 6.5) and much greater than found in *C. remanei* (mean 3.3 (Noble et al., 2021; Parée et al., 2025; Rockman & Kruglyak, 2009; Ross et al., 2011; Snoek et al., 2019; Teterina et al., 2023). These rates reflect the relative physical lengths of the chromosomes: shorter chromosomes typically have more recombination per physical length. In a naïve regression of arm recombination rate on chromosome length, incorporating all autosome arms from the five *Caenorhabditis* species, chromosome length explains 35% of the recombination rate variance (p<10^−5^). The relationship between rate and length is also influenced by the proportion of chromosome with crossover suppression in the center, which varies among species and chromosomes. *C. becei* autosomes have generally typical center sizes, representing approximately half the chromosome length, with the exception of the unusually long center of chromosome V (63%). However, the degree of crossover suppression in *C. becei* is unusually strong, with estimated recombination rates of only 0.1 to 0.6 cM/Mb, lower than that observed in other species (Table S3).

Given findings from artificially lengthened *C. elegans* chromosomes (Libuda et al., 2013), the large size of the *C. becei* X chromosome may exceed the length over which interference acts, allowing for occasional double crossovers. The observed number of crossovers per X chromosome (0.86) is indeed greater than the number expected under complete interference with our cross design (0.75), though not significantly so (*p* = 0.09). On the other hand, the autosomes have fewer crossovers per chromosome (0.99–1.13) than expected (1.25), and the deficit is significant for chromosomes II, III, and IV (2-sided *p*-values < 0.05). The autosomal deficit may be due to segregation distortion (see below) or to direct selection against recombinant chromosomes. In the absence of these forces, the ratio of X to autosome crossovers that we observe is improbable (*p* = 0.0015) if crossover interference on the X is complete. The data are thus equivocal about the possibility of double crossovers on the X, and our advanced intercross experimental design precludes simply counting crossovers to check. Regardless, the *C. becei* X experiences a relatively low absolute recombination rate, due to its unusual physical length and its hemizygosity in males.

### Autosomal sex linkage

*Caenorhabditis* nematodes exhibit a remarkable pattern of correlated segregation of sex chromosomes and autosomal insertion-deletion polymorphisms (T. S. Le et al., 2017; Wang et al., 2010). Males in these species have an X0 sex chromosome composition, and autosomal insertions tend to segregate away from the X chromosome and into X-nullosomic sperm. This pattern, known as skew, has been observed in several Elegans-group species and one more distantly related species, *C. portoensis*, but has not yet been examined in the Japonica group.

We generated an autosomally integrated transgene, *qgIs7*, which drives strong expression of fluorescent reporters. We tested whether *qgIs7* segregated at equal frequencies into male and female progeny of an F_1_ male crossed to a wild-type female. The genetic background of all strains is QG2082, and strains are expected to differ only in the presence or absence of the multicopy transgene array on an autosome. Under the skew model, the transgene should disproportionately segregate into sperm that lack an X chromosome, and therefore it will occur in an excess of male offspring and a deficit of female offspring. We observed this pattern (1,719 offspring, Fisher’s Exact Test *p* = 0.0004). The Transmission Bias Ratio (Le et al., 2017), the ratio of preferred to unpreferred segregation, is 1.19, similar to values observed in other gonochoristic *Caenorhabditis* and much lower than in the selfing species. Another way to state these results is that the autosomal insertion is genetically linked to the nullosome at a distance of 45.7±2.4 cM.

### Segregation distortion due to *Medea* elements

**Figure 4.**
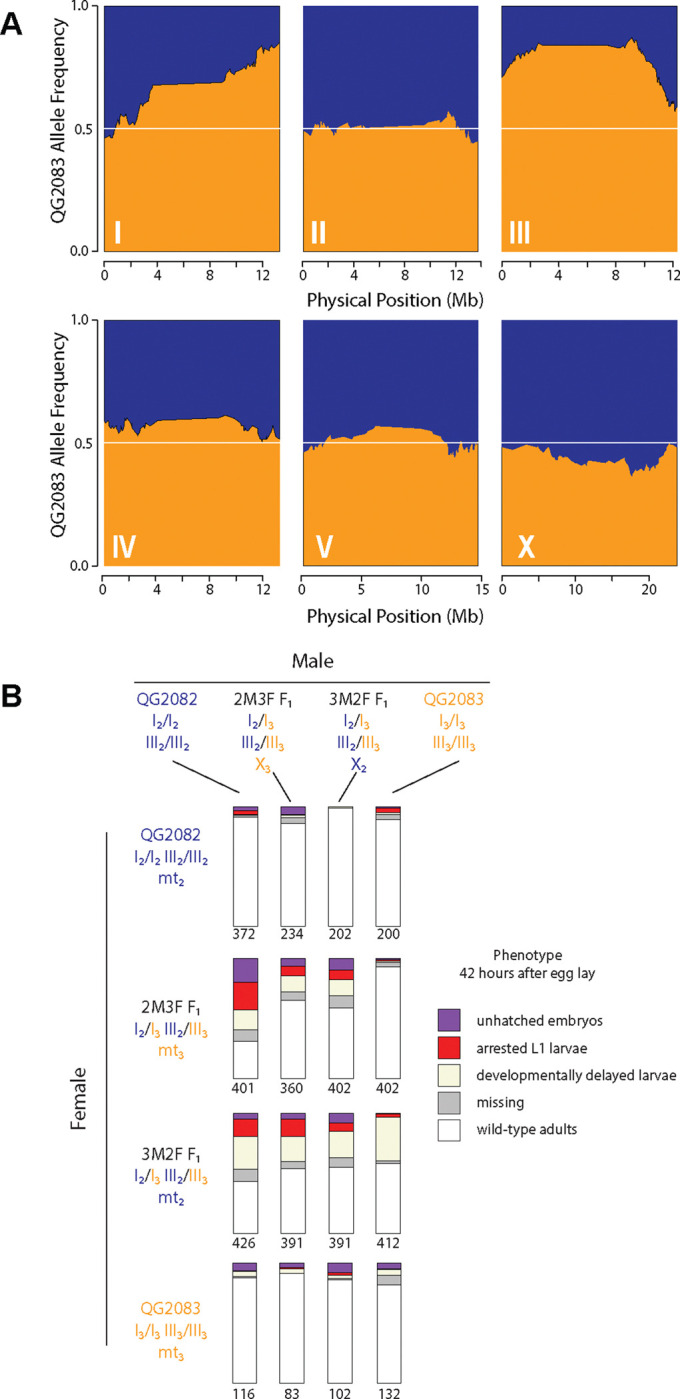
*Medea* alleles cause segregation distortion. **A.** Allele frequencies among G_4_BC_2_ recombinant genomes. Strong distortion of inheritance favoring QG2083 is present on chromosomes I and III. **B.** Experimental crosses implicate maternal-effect loci that act in heterozygous mothers and cause lethality or arrest in homozygous offspring. The phenotype frequencies of progeny from each of 16 classes of cross are shown in stacked bar charts, with sample size below. Parental genotypes on chromosomes I, III, X, and mitochondrial genomes are colored according to their origins in QG2082 or QG2083, and also indicated by the subscripts 2 or 3, respectively.

The genetic mapping experiment revealed strong allele frequency skews on two chromosomes, I and III, with the QG2083 allele having increased in frequency in both cases (Figure 4A). The simplest model for this pattern is selection favoring alleles that increase developmental rate or brood size. However, QG2082 exhibits both faster development and larger brood sizes than QG2083, implying that selection favoring QG2083 alleles may have a more complicated basis. In all three selfing *Caenorhabditis* species, transmission-ratio distortion observed in experimental mapping crosses is attributable to parent-by-offspring genetic interactions, where a heterozygous parent (mother for *Medea* elements, father for *Peel* elements) damages offspring that are homozygous for the non-*Medea/Peel* allele (Ben-David et al., 2017, 2021; Noble et al., 2021; Rockman, 2025; Seidel et al., 2008; Zdraljevic et al., 2024).

We performed experimental crosses to test for *Medea* or *Peel* activity, and we discovered that QG2083 carries alleles with *Medea* activity (Figure 4B). Only offspring of heterozygous mothers were affected, and the affected offspring were in proportion to the expected fraction homozygous for QG2082 alleles (Figure S3). Offspring of F_1_ females x QG2082 males were most severely affected, with less than half of progeny showing wild-type development, while the reciprocal crosses, QG2082 females x F_1_ males, showed no impact. By comparing the counts of affected offspring from different classes of crosses, we estimate that the loci on chromosomes I and III are each acting as *Medea* loci with average penetrances exceeding 50%. The data also show a potential interaction with mitochondrial genotype or a parent-of-origin effect, as also observed for *Medea* elements in *C. tropicalis* (Ben-David et al., 2017, 2021; Noble et al., 2021), seen in the different patterns in the second and third rows of Figure 4B.

### Repetitive Element Landscape

The genome of *C. becei* has a low repetitive element content (15%), among the lowest in *Caenorhabditis*, comparable to *C. niphades* (11.3%), *C. bovis* (13%), and *C. elegans* (18%) (Sun et al., 2022; Woodruff & Teterina, 2020). The most abundant repeats were Tc1-Mariners (6.6%), consistent with their dominance across other *Caenorhabditis* species (Woodruff & Teterina, 2020). Other abundant repeat types included CACTA elements (1.3%), simple repeats (1.1%), and unclassified repeats (4.2%) (Figure S4).

Repetitive elements were more abundant in the arms than in the centers (Figure S5). This non-random distribution of repeats is consistent across most *Caenorhabditis* species, except for *C. inopinata* and *C. bovis*, where a more homogeneous repeat landscape is observed due to the recent expansion of Tc1-Mariner elements (Woodruff & Teterina, 2020).

To estimate repetitive element age, we used Kimura distances as a proxy for divergence. Repeat divergence distributions showed no peaks indicative of recent transposon expansion, and we found no evidence of recent repetitive element activity for any repeat type.

### Gene structure

We used two experimental strategies to generate mRNA data for gene annotation. First, we grew mixed-stage mixed-sex populations under five divergent conditions then pooled the worms, extracted RNA, and Illumina-sequenced a stranded mRNA library. Second, we grew mixed-stage mixed-sex populations under two conditions, isolated RNA, and generated long-read Iso-seq data. We integrated these data with protein sequence data from other species to infer gene models.

We annotated 24,025 protein-coding genes, and 32.81% of the genome is CDS. We have not annotated non-coding RNAs, though they are likely to be interesting in this species. *C. becei* lacks the highly conserved miRNA *let-7* and may be using other mechanisms to replace its function (Nelson and Ambros 2021).

In *C. becei*, introns and coding sequences (CDS) have very different proportions in chromosomal arms and centers: arms are heavily enriched for intronic DNA, while centers are dense with CDS (Figure S6). In *C. niphades*, *C. becei*’s closest relative with a published chromosomal assembly, introns and CDS are similarly extensive in the arms, but intronic DNA decreases and CDS increases in the centers (Figure S6).

### Genomic GC Content

**Figure 5.**
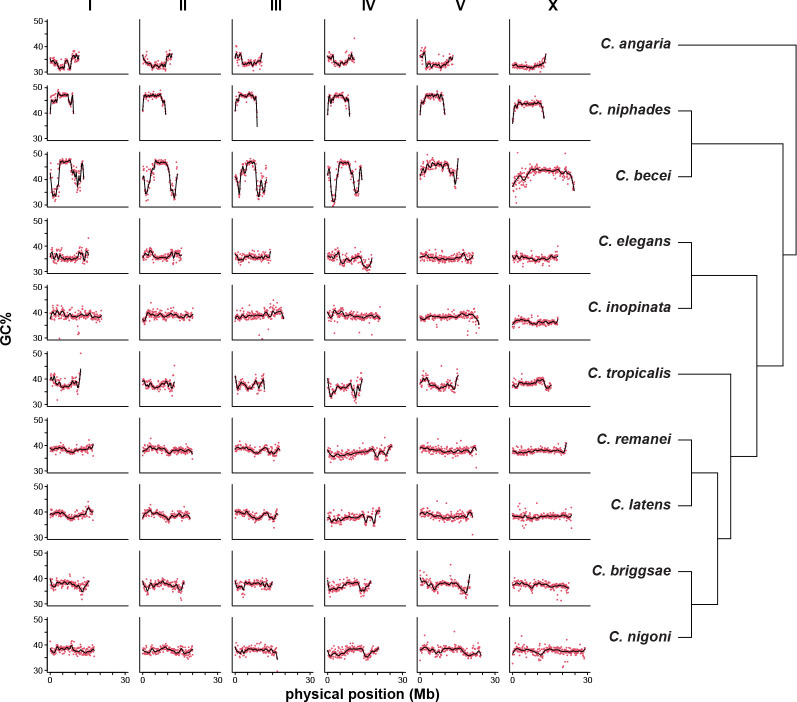
GC content varies along chromosomes and among species. GC% is plotted for non-overlapping 100kb windows along the length of each chromosome, along with loess-fitted lines (degree 2, span 1/8). *C. becei* is unusual in its high GC content (a trait shared with *C. niphades*, the other Japonica Group species with a chromosomal assembly) and its high variance, most notably on chromosomes I-IV.

Among species with chromosomal assemblies, *C. becei* has genomic GC content that is both high and unusually variable along the chromosomes (Figure 5). The variance in GC content along chromosomes is much greater in *C. becei* than in the other species with chromosomal assemblies, independent of the scale over which GC% is measured (Figure S7). In *C. becei*, chromosomes I-IV exhibit notably high GC content in their centers and lower GC content in their arms. In contrast, chromosomes V and X show a more uniform distribution of high GC content along both the center and the arms.

**Figure 6.**
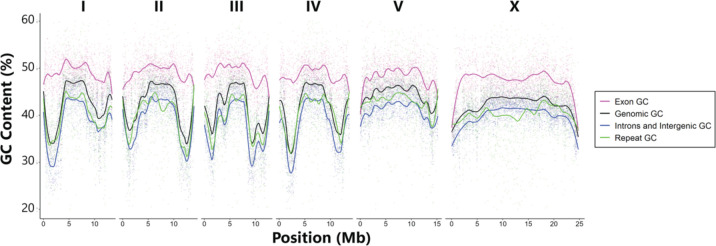
GC content of genomic features in *C. becei*. GC content was calculated from the counts of G+C divided by the total number of bases of the feature within non-overlapping 10 kb windows along the length of the chromosome with LOESS-fitted lines (span = 0.2).

In *C. becei*, the average GC content of features follows a descending order of exon > genomic average > repeat > introns/intergenic regions (Figure 6). This ordering is conserved across species, but the differences are more pronounced in *C. becei* and Elegans Group species than in *C. niphades* or *C. angaria* (Figure S8). In these latter species, GC content is relatively consistent across the chromosomes within each species and across annotation features.

### Codon and Amino Acid Usage

**Figure 7.**
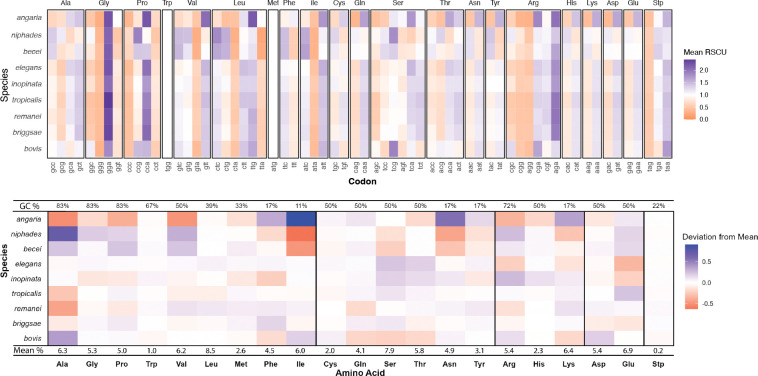
Codon usage and amino acid composition in *Caenorhabditis* species. **A.** Relative synonymous codon usage (RSCU). Rows correspond to species, and columns represent codons grouped by amino acid. Tiles represent the RSCU value for each codon in a given species. RSCU values greater than 1.0 indicate codons used more frequently than expected under uniform usage, with higher values shaded in orange and lower values shaded in purple. **B.** Amino acid usage: Rows correspond to species, with the last row displaying the mean percentage of each amino acid across all species. The mean is calculated by averaging the percentage of each amino acid in the CDS across species. Tiles represent the deviation from the mean amino acid percentage for each species, with the mean calculated separately for each amino acid. Fill colors indicate the direction and magnitude of the deviation: values above the mean are shaded in blue, and values below the mean in red. The intensity of the shading reflects the magnitude of the deviation. The top row shows the average GC% of codons encoding each amino acid.

Distinct patterns of codon usage are observed among *Caenorhabditis* species, potentially reflecting GC content biases. *C. becei* and *C. niphades* have high GC content and use codons with higher GC content, while *C. angaria* has lower GC content and uses codons with lower GC content (Figure 7). Species in the Elegans group exhibit intermediate codon usage patterns, showing less pronounced deviations compared to the Japonica and Drosophilae groups.

A similar trend is observed in amino acid usage, in which *C. becei* and *C. niphades* show positive deviations from the mean for amino acids associated with high GC codons and negative deviations for those linked to low GC codons. In contrast, *C. angaria* exhibits the opposite pattern, with positive deviations for amino acids associated with low GC codons and negative deviations for those linked to high GC codons (Figure 7B). These results suggest that GC content influences both codon and amino acid usage across *Caenorhabditis* species.

### Synteny

**Figure 8.**
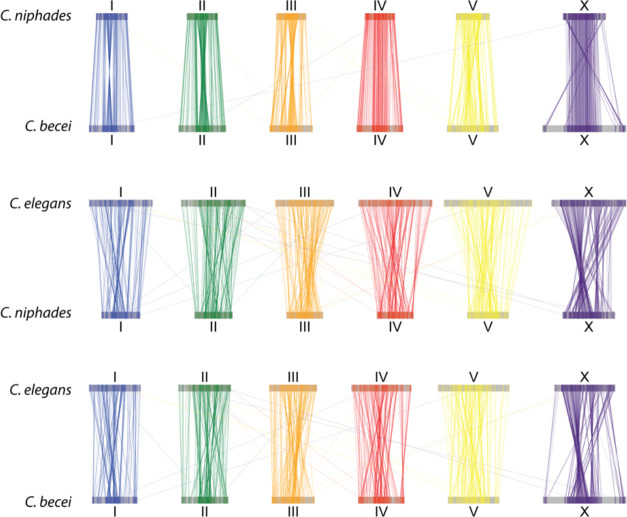
Synteny between *C. elegans*, *C. becei,* and *C. niphades*. Each line connects single-copy orthologues (n=1878).

We observe strong synteny between the chromosomes of *C. becei* and *C. elegans* (Figure 8), as expected given the well-established conservation of ancestral linkage groups (Nigon elements) in Rhabditid nematodes (Gonzalez de la Rosa et al., 2021). The most striking feature of the synteny pattern is the absence of 1:1 orthologs in the arms of the *C. becei* X chromosome. The large size of the X is associated with a sizable increase in gene number on the X chromosome: 5,345 in *C. becei* compared to 2,877 in *C. elegans*.

We compared synteny between *C. becei* and *C. niphades,* the closest species with a chromosomal assembly, using a set of 1,878 one-to-one orthologs. We found extensive collinearity across all chromosomes, though key differences remained. On Chromosome X, the *C. niphades* ends are inverted relative to *C. becei* (equivalently: the chromosomes differ by a giant inversion). The regions that lack single-copy orthologs between *C. becei* and *C. elegans* also lack single-copy orthologs in the smaller *C. niphades* X chromosome. This suggests that the gene duplication on *C. becei* Chromosome X is a recent, lineage-specific expansion rather than a shared feature of the Japonica group (Figure 8). For Chromosomes I, II, III, and V, inversions were restricted to the central regions, while the arms remained highly collinear.

### Comparative ortholog analysis reveals widespread gene duplications in *C. becei*

The gross similarities between the *C. becei* and *C. niphades* genomes allow us to probe the major differences we have identified between them: *C. becei* has a bigger genome, with more heterogeneity in GC and feature distribution, and its X has large regions that lack one-to-one orthologs. To understand how gene family evolution contributes to these patterns, we used gene annotations from eight high-quality chromosomal assemblies (*C. angaria, C. becei, C. briggsae, C. elegans, C. inopinata, C. niphades, C. remanei,* and *C. tropicalis*) to classify genes into orthogroups. Orthogroups were categorized as single-copy, multi-copy, or unassigned. Multi-copy orthogroups contained more than one gene within a species, while single-copy orthogroups included genes that were present as a single copy in a given species but had orthologs in other species. Unassigned orthogroups consisted of genes that were species-specific, with no detectable orthologs in other species.

**Figure 9.**
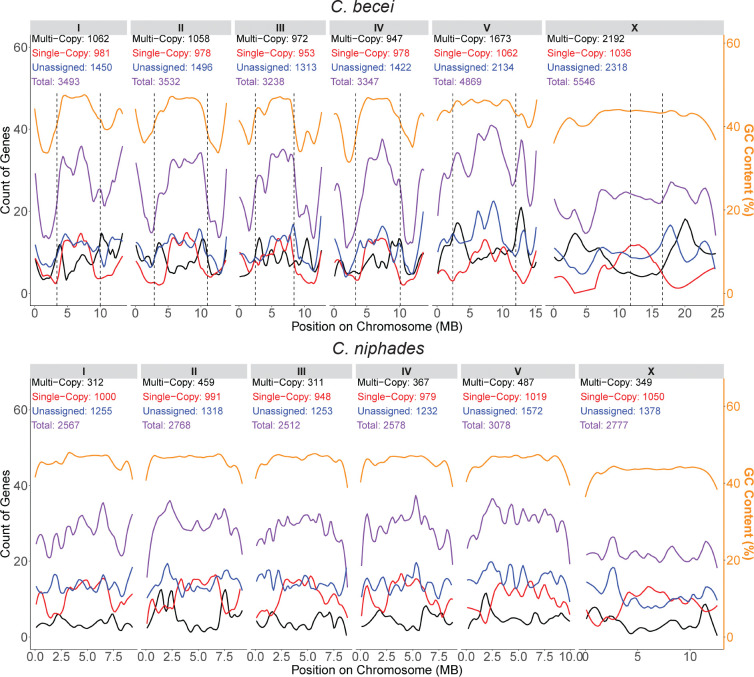
Distribution of duplicated, single-copy, and unassigned genes across the chromosomes of *C. becei* and *C. niphades*. Gene counts are plotted in 100 kb windows across the chromosomes of each species, with LOESS-fitted lines (span = 0.2). Genes are categorized based on orthogroup analysis: multi-copy (black), single-copy (red), and unassigned genes (blue). Multi-copy genes belong to orthogroups with more than one gene in a species. Single-copy genes belong to orthogroups with only one gene in a species. Unassigned genes are single-copy genes that are not assigned to any orthogroup shared with another species. GC content (gold) are the percentage of bases within a 100 kb window that are G+C, with LOESS-fitted lines (span = 0.2). Chromosome arm-center boundaries are plotted as dotted lines.

Single-copy genes were similar in number, distribution, and length across the chromosomes of both *C. becei* and *C. niphades*, with approximately 1,000 single-copy genes per chromosome, spanning an average length of 3.45 ± 0.24 Mb per chromosome in *C. becei* and 3.09 ± 0.29 Mb in *C. niphades*. However, the relative proportion of genome occupied by single-copy genes was lower in *C. becei,* because multi-copy orthogroups occupy a much larger proportion of its genome (Figure S9). In both species, single-copy genes were most abundant in chromosome centers, less frequent in the arms, and slightly enriched at chromosome tips, following a consistent pattern across all chromosomes (Figure 9).

Patterns for multi-copy genes differed among *C. becei* chromosomes, just as they do for GC content. Chromosomes I–IV are similar in their counts of multi-copy genes (~1000), while chromosome V and X have substantially more: 1,673 and 2,192 (Figure 9). In contrast, *C. niphades* has fewer multi-copy genes, ranging from 311 to 487 per chromosome (Figure 9B). In *C. becei*, multi-copy genes tend to be abundant in the transition regions between the arms and centers.

The exceptionalism of *C. becei* chromosomes V and X extends to the unassigned (species-specific) orthogroups as well. The number of unclassified genes per chromosome is comparable between *C. niphades* and chromosomes I-IV of *C. becei*, while chromosomes V and X in *C. becei* carry about 60% more (Figure 9).

Overall, comparing the same chromosome between species, chromosomes I-IV differ proportionately in the fraction dedicated to single-copy and unassigned orthogroups and intergenic regions, all of which are longer in *C. becei* than in *C. niphades*, while each *C. becei* chromosome also has extra length due to much higher numbers of genes from multi-copy orthogroups. On *C. becei* chromosomes V and X, the contributions of unassigned and multi-copy orthogroups are magnified. While the distribution of genes along the chromosomes matches the single-copy distribution on chromosomes I-IV, the pattern on V and X deviates from the single-copy gene distribution due to the higher abundance of multi-copy and unassigned genes in the arms.

Within *C. becei*’s large X chromosome, genes belonging to orthogroups with the highest gene counts are often clustered, indicating localized expansions or tandem duplications within these gene families (Figure S10). The duplication patterns on chromosome X involve multiple orthogroups, rather than being dominated by a single expanding gene family (Table S4).

Functional analysis of the X-chromosome orthogroups identified specific common protein domains. These include “T-box transcription factor, DNA-binding domain,” indicating roles in transcriptional regulation, and “RNA exonuclease REXO1/REXO3/REXO4-like” domains, suggesting functions in RNA processing and turnover (Table S4). However, many orthogroups lacked functional annotations, highlighting substantial genomic novelty on the X, particularly among expanded gene families (Table S4).

## DISCUSSION

*Caenorhabditis becei* is a promising model for studies of gonochoristic *Caenorhabditis*, and we here lay the genetic and genomic foundations for future studies. Part of its appeal is its type locality, the intensely studied forests of Barro Colorado Island, Panamá. Our phylogenetic analysis finds *C. becei* deeply nested within a neotropical radiation of species in the Japonica Group, with close relatives spread across Central and South America and the Caribbean.

The *Caenorhabditis* clade provides a powerful lens into genome evolution, recombination, and species divergence, but most studies have focused on the Elegans group. Draft genomes from its sister, the Japonica group, have revealed generally high GC content and compact genomes (Stevens et al., 2019; Sun et al., 2022). *C. becei* now joins *C. niphades* in representing the Japonica group with chromosomally contiguous assemblies. The *C. becei* genome is much larger than that of *C. niphades*, but the two remain highly syntenic and collinear for one-to-one orthologs, suggesting that genome expansion in *C. becei* is a recent event rather than a shared feature of the *Japonica* group.

The structure of the *C. becei* genetic map, the first for a *Caenorhabditis* species outside the Elegans group, is very similar to that observed in the four species with available maps (Barnes et al., 1995; Noble et al., 2021; Rockman & Kruglyak, 2009; Ross et al., 2011; Stevens et al., 2022; Teterina et al., 2023). Recombination is structured into relatively discrete domains, with low recombination in the centers and high recombination in the arms. The most conspicuous departure from other species is the large extent of the low-recombination center on chromosome V, spanning 63% of the chromosome. Another familiar feature of *Caenorhabditis* genetics is also conserved: the skewed inheritance of autosomal indels with respect to segregation of the X chromosome in male meiosis, which we demonstrated using new strains carrying autosomally integrated fluorescent transgenes.

Our genetic map, like those previously generated for the androdioecious *Caenorhabditis* species, was affected by strong segregation distortion on several chromosomes. We showed that the observed segregation distortion likely reflects the action of multiple *Medea* elements, which act in heterozygous mothers and cause developmental delays or lethality in offspring that do not inherit them (Beeman et al., 1992). This is the first demonstration of *Medea* elements in the Japonica group and in dioecious *Caenorhabditis*, and it suggests that the striking ubiquity of these genetic elements is a broadly shared feature of *Caenorhabditis* biology (Ben-David et al., 2017, 2021; Noble et al., 2021; Seidel et al., 2008; Zdraljevic et al., 2024), and not a quirk of the androdioecious mating system. In our study, the alternate alleles at the *Medea* loci are from isofemale lines collected within a few kilometers of one another in 2012, and their evolutionary dynamics are likely to depart substantially from those predicted for primarily selfing lineages (Rockman 2025).

At the DNA sequence level, the *C. becei* genome is distinctive for its high GC content, a feature shared with *C. niphades*. The elevated GC content influences codon usage bias in *C. becei*, as demonstrated by comparative analysis across *Caenorhabditis*: species with higher genomic GC content tend to preferentially use GC-rich codons, a pattern observed in both *C. becei* and *C. niphades* (Figure 7). This bias extends beyond codon preferences to amino acid composition: both *C. becei* and *C. niphades* preferentially use amino acids encoded by GC-rich codons.

Chromosomes I-IV (but not V or X) have dramatic heterogeneity in GC content, very high in centers and tips and very low in the chromosome arms. These arm regions are also dramatically depleted of coding sequences relative to other parts of the genome (Figure S6), and coding sequences are substantially more GC-rich than other classes of sequence. However, the pattern is not driven solely by coding sequence density, as the intronic, intergenic, and repetitive classes of sequence have high GC content outside of the low-GC arms (Figure 6). The extreme variation in GC content along chromosomes I-IV may reflect chromosome-specific mutational processes.

Chromosomes V and X differ from the others. They have much more uniform GC content (Figures 5 & 6) and also a more uniform density of coding sequences (Figure S6), in part due to an unusually high number of gene duplications and orphan genes (Figure 9). Orphan genes, which lack detectable homologs in other species, may arise through *de novo* evolution, rapid divergence, or duplication followed by functional divergence (Tautz & Domazet-Lošo, 2011). Their enrichment on chromosomes V and X suggests that these chromosomes may act as hotspots for lineage-specific gene emergence (Bouvarel et al., 2024).

Chromosomes V and X are also distinctive for their low recombination rates, due to both long physical lengths, a long central domain on V, and X hemizygosity in males. These features correlate with the high number of gene duplications and orphan genes. Gene duplications are most abundant around the arm-center boundaries on chromosome V and in the chromosome arms on the X (Figure 9). The absence of large-scale duplications in chromosome centers does not imply that duplications never arise there but suggests that they may be less likely to be retained. This may be due to strong purifying selection acting on functionally constrained single-copy genes and orphan genes, which dominate these regions. Orphan genes persist in chromosome centers, perhaps via functional integration into essential pathways (Tautz & Domazet-Lošo, 2011).

Repeat elements are strongly enriched in chromosome arms and depleted in centers, matching recombination rate variation across the genome. The depletion of repeats in chromosome centers is likely due to strong purifying selection, which removes insertions in these functionally constrained regions (Woodruff & Teterina, 2020). This is similar to the absence of gene duplications in centers, where functionally constrained genes dominate, and disruptive insertions are more likely to be deleterious (Thomas, 2006). The arm-rich, center-poor repeat distribution is also observed in other *Caenorhabditis* species without recent repeat activity (Woodruff & Teterina, 2020).

The *C. becei* X chromosome is exceptionally large, 40% longer than that of *C. elegans* (Figure 2) and contains more genes than any other chromosome (Figure 9). The expansion of the X in *C. becei* suggests unique evolutionary pressures, perhaps involving reduced recombination, sex-specific selection, and dosage-sensitive gene accumulation. Because the X is hemizygous in males, it experiences distinct selection pressures (Cutter, 2018). The absence of a homologous pairing partner prevents recombination in males, reducing the efficiency of purging deleterious mutations while exposing recessive variants to stronger selection. This could result in greater retention of beneficial mutations, including duplicated and orphan genes, if they provide an adaptive advantage (Maciejowski et al., 2005).

The accumulation of duplicated genes in *C. becei* raises questions about their evolutionary significance and functional relevance. In *C. becei*, the clustering of gene expansions within orthogroups suggests non-random duplication events, possibly linked to selection for co-regulated gene networks or dosage effects (Csankovszki et al., 2004; Maciejowski et al., 2005; Strome et al., 2014). Functional analyses of X-linked duplicated genes revealed an overrepresentation of transcription factors and RNA-processing genes, suggesting that at least some of these duplications may play regulatory roles.

The *C. becei* genome represents an appealing model for studies of genome evolution, with interactions between gene duplication, GC content heterogeneity, recombination rate variation, and codon usage biases. The nonrandom chromosomal distribution of gene duplications and orphan genes suggests that selection, recombination, mutation, and genome architecture play key roles in shaping gene retention. Future studies should focus on determining the functional significance of orphan and unclassified genes, particularly those located in chromosome centers and X-linked regions, to assess whether they contribute to adaptive processes, dosage compensation mechanisms, or reproductive functions. Understanding the role of recombination in shaping chromosomal architecture will also be critical, particularly in regions where gene duplications and transposons appear to be accumulating. Comparative analyses with additional *Japonica* species will provide further insight into which of these patterns are unique to *C. becei* and which reflect broader evolutionary trends within the clade.

## METHODS

### Phylogeny

We collected protein sequence data for 57 species from published genomes and transcriptomes and, by permission, from the *Caenorhabditis* Genome Project (caenorhabditis.org). In addition, we generated new data for three undescribed species from Queensland, Australia (*C. sp. 51*, *C. sp. 52*, and *C. sp 67*), using RNAseq and *de novo* transcriptome assembly, exactly as described for *C. krikudae* in Sloat et al. (2022). The fastqs and transcriptome assemblies are deposited with NCBI in association with BioProject ID PRJNA1128046. The sources of data for all 60 species are provided in Table S5.

To identify a set of single-copy orthologs for phylogenetic analysis, we used BUSCO 5.3.0, (Seppey et al., 2019), using the nematode_odb10 dataset on each of the protein fasta files. For each protein sequence in its database, BUSCO identifies homologous sequences in the input file and classifies them as single or multicopy. We identified the set of inferred single-copy orthologs present in at least 80% of the species in the dataset and generated multisequence fasta files for each, using *busco2fasta* (https://github.com/lstevens17/busco2fasta). We then used MAFFT 7.475 (Katoh & Standley, 2013) to align the sequences, with default settings. We trimmed these alignments to eliminate poorly aligned regions using TrimAl 1.4.1 (Capella-Gutiérrez et al., 2009) with settings -gt 0.8 -st 0.001 -resoverlap 0.75 -seqoverlap 80. This process yielded 2,059 alignments. We estimated maximum-likelihood gene trees using IQ-TREE 1.6.12 (Nguyen et al., 2015) with the LG+I+G model (Le & Gascuel, 2008; Yang, 1994). We then estimated a species tree from the collection of gene trees under the coalescent model implemented in ASTRAL-III 5.7.8 (Zhang et al., 2018). To estimate branchlengths, we used a concatenation of the 2,059 sequence alignments (806,191 amino acid positions), generated with catfasta2phyml (https://github.com/nylander/catfasta2phyml) to concatenate the alignments. We then IQ-TREE with the LG+I+G model to fit branch lengths to the ASTRAL tree. We rooted the phylogeny using the Auriculariae Group as the outgroup to the rest of *Caenorhabditis* (Sloat et al., 2022). Plots were generated using the *ape* package (Paradis & Schliep, 2019) in R (R Core Team, 2024).

### Genome Assembly

QG2082 was grown on NGMA plates at 25°. A mixed-stage mixed-sex population was flash frozen in liquid nitrogen and the frozen worm pellet was shipped to the University of Oregon Genome and Cell Characterization Core Facility for HiFi sequencing. Flash-frozen worms were also shipped to Dovetail Genomics (Santa Cruz, CA; https://dovetailgenomics.com) for Hi-C sequencing with 150bp Illumina reads.

Short-read data for QG2082 (Illumina paired-end), QG2083 (Illumina paired-end and mate-paired), and for G_4_BC_2_ lines (Illumina paired-end) are described in Lamelza et al. (2019).

We generated assemblies using PacBio HiFi reads (≥Q20) with assemblers Flye (Kolmogorov et al., 2019), Hifiasm (Cheng et al., 2021), and Hi-Canu (Nurk et al., 2020), and Hi-C-scaffolded Flye and Hifiasm assemblies with Juicer (Durand et al., 2016). As in Noble et al. (2021), we then evaluated each assembly for consistency with genetic linkage data (implemented in a Snakemake pipeline available at https://github.com/lukemn/becei), and then closed gaps of estimated 0cM genetic distance where strand-consistent spanning reads from local mapping were available. The final assembly drew partially redundant sequences from two primary assemblies (Flye -m 10 -g 100Mb; Hifiasm -l 0). For chromosomes I, IV and X, sequences from Hi-C scaffolding of the Hifiasm assembly were used to span primary sequences. Each chromosome was iteratively polished with HiFi reads, using calls from DeepVariant (Poplin et al., 2018) and bcftools norm/consensus (Danacek et al., 2021), until either no homozygous variants were called, or a local minimum in read mapping error rate was found. Telomeric sequences were counted using R library *seqinr* (Charif & Lobry 2007).

To assemble the *C. becei* mitochondrial genome, we first mapped Q20- and Q30-filtered PacBio HiFi reads to the *C. becei* nuclear reference genome using minimap2 (version 2.22; Li 2018), and extracted unmapped reads using samtools (version 1.14; Danecek et al., 2021). We then aligned unmapped Q20 reads to the mitochondrial genome of *C. nouraguensis* (Accession: NC_035250.1), using minimap2 to identify mitochondrial reads. Subsequently, we *de novo* assembled the Q20 mitochondrial reads using Flye (version 2.9.5; Kolmogorov et al., 2019) with a minimum overlap of 1,000 bp, and generated a single circular 13,708 bp contig with high coverage. This draft contig was then used as a reference to map the higher-accuracy Q30 HiFi reads, which we assembled using Flye with a 10 kb minimum overlap, producing a single high-confidence circular contig. This final assembly was annotated using MITOS2 (Al Arab et al., 2017; Donath et al. 2019) and manually curated.

The nuclear and mitochondrial genome assemblies and underlying data are available from the NCBI at BioProject PRJNA989223.

### Genetic Map

Data for genetic map construction are described in Lamelza et al. (2019). For the map described here, we used short-read sequence data from 92 individual G_4_BC_2_ populations. Each such population is a pool of worms carrying one unique set of recombinant chromosomes, each the product of several generations of meiosis. Reads were mapped to the QG2082 reference genome and ancestries inferred along each chromosome using MSG (Andolfatto et al., 2011). The data plotted in Figure 3A are in Table S2.

Domain boundary positions for *C. becei* and other species were estimated by segmented linear regression using the *segmented* package (Muggeo, 2008). We first interpolated genetic positions for 200 evenly spaced physical positions along each chromosome, then excluded 1 Mb from each end of each chromosome to avoid effects of tip regions, which generally have suppressed recombination and whose boundaries are poorly resolved in most species given the limited available data. We then regressed genetic position on physical position with the constraint that there be three linear segments. Note that domain boundary positions are imprecise, given the modest number of crossovers present in the data; as shown by simulations in (Rockman & Kruglyak, 2009), even in the case of precise boundaries, uniform domains, and relatively large samples of crossovers, the confidence intervals for boundaries are on the order of a megabase.

To generate null distributions for crossover counts under the hypothesis of complete interference, we simulated G_4_BC_2_ crosses in R (File S1).

For comparisons with other species (Table S3 and Figure S2), we used the following datasets:

*C. briggsae* 99 F_2_ RILs QX1410 × VX34 Stevens et al., 2022 https://github.com/AndersenLab/briggsae_reference_genome_MS/blob/main/3_recombinationmap/CB_genetic_map.Rda

*C. tropicalis* 119 F_2_ RILs NIC58xJU1373 Noble et al., 2021 https://github.com/lukemn/tropicalis/blob/master/geneticMap/data/c_tropicalis_flye_geneticMap.rda

*C. remanei* 341 F_2_s PX506 × PX553 Teterina et al., 2023 https://figshare.com/ndownloader/files/41808561

*C. elegans* 1045 RIAILs CB4856 × N2 & CB4856 × QX1430 (N2 *peel-1(ttTi12715)* I *npr-1(qg1)* X) Brady et al., 2019 https://github.com/AndersenLab/linkagemapping

The X-chromosome map was re-estimated after excluding markers surrounding the *npr-1* introgression *qg1* in RIAIL sets 2 and 3, which cause a spurious map expansion otherwise.

*Pristionchus pacificus* 93 F2s PS312 × PS1843 Rillo-Bohn et al., 2021 https://cdn.elifesciences.org/articles/70990/elife-70990-fig8-data1-v2.xlsx

We used the *P. pacificus* oocyte genetic map data only, due to strain-dependent crossover suppression in the male data, likely attributable to inversions. The X data are also distorted in a putatively strain-dependent fashion, as described in Rillo-Bohn et al. (2021)

### Autosomal Sex Linkage

To create a strain with an integrated transgene insertion, we used Gibson cloning to generate plasmids pSAS02 (*Cbr-P*_*plg-1*_*::GFPnovo2::unc54utr* with the pSM-GFPnovo2 backbone) and pSAS06 (*Cel-P*_*fkh-6*_*::mCherry::unc54utr* in the pCFJ104 backbone). The backbone plasmids were acquired from Addgene and the final plasmids were verified by Oxford nanopore whole-plasmid sequencing (Plasmidsaurus.com). We PCR-amplified the reporters (*promoter::FP::UTR*) from the plasmids and injected them together into QG2082 and recovered lines transmitting both transgenes. Several hundred L4 animals of a transmitting line were UV irradiated at 500 J/m^2^. Those animals were passaged for 5 generations before backcrossing individuals to QG2082 to screen for Mendelian segregation of the integrated array. Lines positive for integration were backcrossed an additional 12x before homozygosing the integrated array as line QG4602 (*qgIs7*). Plasmid sequences are provided in File S2.

To test for skew, we performed reciprocal crosses between QG2082 and QG4602 under standard conditions at 25°C. We then crossed F_1_ males to QG2082 females and collected and scored all progeny laid within timed windows. We observed 343 insertion^+^ males, 278 insertion^−^ males, 507 insertion^+^ females, and 591 insertion^−^ females. The biased sex ratios are consistent with the known bias in *C. becei* (Huang et al., 2023). Recombination frequency is estimated as the number of unpreferred-phase gametes (X+insertion or 0+wildtype) out of *n* = 1,719 total, and the 95% confidence interval is the normal approximation for binomial proportions.

### Segregation distortion

To test for parental-by-offspring genetic interactions (*Medea* or *Peel*), we performed replicates of the sixteen possible pairwise crosses between four genotypes: QG2082, QG2083, and the F_1_s from each direction of the cross between QG2082 and QG2083. We included all possible F_1_s in order to account for possible effects of mt-DNA or X-linked interactors. The reciprocal F_1_ females differ in their mitochondrial genotype, and the reciprocal F_1_ males differ in which X chromosome they carry. Under a *Medea* hypothesis, the only affected individuals will be a subset of the progeny of heterozygous females, specifically those offspring homozygous for QG2082 alleles on the right arm of chromosome I or the center of chromosome III. Under a *Peel* hypothesis, the only affected individuals will be the comparable subset of the progeny of heterozygous males. A detailed accounting of the expected results under the *Medea* hypothesis are shown in Figure S3.

Worms were maintained on OP50 *E. coli* at 25°C during the experiments and for three generations beforehand. For each cross, 9 L4 females and 10 L4 males were transferred to a 6cm plate on day 1 and left to mature and mate overnight. The following day (day 2), females with mating plugs were transferred to individual plates for a 7-hour egg lay period. Crosses were handled in a random order and plates were blinded to genotype for subsequent scoring. After the egg lay, females were then removed and the embryos laid on the plate were counted. On day 3, 18 hours after the end of the egg lay, unhatched embryos and deformed, arrested L1 larvae were counted. The deformed larvae were characteristically arrested in a two- or three-fold posture as if unable to move after hatching. On day 4, 42–48 hours after the end of the egg lay period, wild-type males and females, L4 males and females, and active L1-L3 larvae were counted. At this timepoint all larvae are the product of the original egg lay, as eggs laid by the adult offspring have not yet hatched. Raw count data are reported in Table S6.

To summarize the results, counts were pooled by cross and plotted as proportions, with the total being the number of embryos counted on day 2, immediately after the egg lay. In many cases, the sum of unhatched embryos, deformed L1s, developmentally delayed larvae, and wild-type adults was less than the number of embryos present initially. This gap is due in part to the difficulty in observing the small arrested L1 larvae, but it also reflects slight errors in counting each of the classes of embryos and worms. This gap between total embryos counted on day 2 and total offspring accounted for on days 3 and 4 is recorded as “missing” in Figure 4.

Under the two-*Medea* model, a greater proportion of QG2082-backcross progeny will be affected than F_2_ progeny. More specifically, assuming the *Medeas* have independent (i.e, multiplicative) effects, the difference in affected proportions between these two classes of progeny is an estimator of 1/16 *β*_I_ + 1/16 *β*_III_ + 3/16 *β*_I_*β*_III_, where the *β*s are the penetrances of the *Medea* loci on chromosomes I and III. Pooling observations across relevant experiments, we count 517/827 affected offspring in the QG2082 backcrosses and 644/1544 in the F_2_s. The difference in affected proportions is 0.21±0.04 (95% confidence interval calculated via Agresti-Caffo method). If the two *Medeas* have the same penetrance, the data provide an estimate of the penetrance as 0.77±0.10. Alternatively, if one *Medea* is completely penetrant, the data imply the other has a penetrance ~0.59±0.16.

### Repetitive Element Analysis

We generated a redundant set of repetitive element libraries, identified *de novo* using the tools RepeatModeler (Flynn et al., 2020), TransposonPSI (B. Haas, 2007), LTRharvest (Ellinghaus et al., 2008), LTRdigest (Steinbiss et al., 2009), SINE-Scan (Mao & Wang, 2017), SineFinder (Wenke et al., 2011), TirVish (Gremme et al., 2013), HelitronScanner (Xiong et al., 2014), MITE-Tracker (Crescente et al., 2018), MUSTv2 (Ge et al., 2017), and MiteFinderII (Hu et al., 2018). These libraries were merged with nematode-specific repeat libraries from Repbase (Bao et al., 2015) and Dfam (Storer et al., 2021) to create a single redundant repeat library. To remove redundancies, we used USEARCH (Edgar, 2010) to cluster the sequences in the library. The resulting non-redundant library was further filtered for any potential non-repeat clusters by using BlastX (Camacho et al., 2009) to search against nematode-specific proteins and non-coding RNAs.

The repeat discovery pipeline was adapted from a protocol previously used to characterize repeats in the *C. inopinata* genome (Coghlan et al., 2018; Woodruff & Teterina, 2020). To validate this pipeline, we replicated previously reported repeat contents for *C. inopinata*, *C. elegans*, *C. briggsae*, *C. nigoni*, *C. remanei*, and *C. bovis* (Woodruff & Teterina, 2020).

We used the hierarchical classification schema applied in other *Caenorhabditis* species (Woodruff & Teterina, 2020). The non-redundant repetitive element library was classified using multiple tools: RepeatClassifier (Flynn et al., 2020), Dfam Classifier (Storer et al., 2021), TransposonUltimate RFSB Classifier (Riehl et al., 2021), and Geneious Sequence Classifier (https://www.geneious.com). We assigned the consensus classification from these tools, with conflicting classifications labeled as “unknown.”

Repeat libraries were generated independently for each genome assembly, and each genome was masked with RepeatMasker (http://www.repeatmasker.org/), using only their specific repeat libraries. We used the final RepeatMasker-generated masking results to characterize the genomic repeat landscape at both the global and taxonomic levels. To account for nested repeats, we disjoined the GFF so that each base pair in the genome was assigned to only one TE copy. This allowed us to distinguish between the number of repeat insertions and the number of bases covered by a given repeat taxon (Anderson et al., 2019). We estimated the age of repetitive elements by calculating Kimura distances (Kapusta et al., 2017), which measure the divergence of individual repeat copies from their consensus sequence. Kimura distances were extracted from the “.align” file generated by RepeatMasker, and we calculated the mean Kimura distances across non-overlapping 10-kb windows for all repeat taxonomic ranks.

### Gene Structure

QG2082 worms were raised under standard conditions with OP50 *E. coli* food and then split into five conditions, as described in Sloat et al. (2022): 1) CemBio bacterial strains (Dirksen et al., 2020) as food; 2) OP50 and standard conditions; 3) OP50 plus heat stress; 4) OP50 plus cold stress; 5) starvation. Temperature stresses consisted of 35°C or 4°C for 2 hours followed by a 2-hour recovery prior to RNA extraction. Total RNA was isolated using TRIzol following the protocol described in Green & Sambrook (2020). The mRNA libraries were constructed using the Illumina Stranded mRNA Prep Ligation protocol. These barcoded libraries were then pooled and sequenced using a NextSeq 500 MidOutput 2X150 for 300 cycles.

For the Iso-seq RNA extraction, we grew two sets of mixed-stage worms. One was well fed and the other starved for 2 days to generate a mix of dauer, arrested L1s, and starved adults. Worms were washed 5x in M9 to remove bacteria. The resulting pellet was resuspended in a mortar and pestle tube with 100 μL of TriZol and flash frozen in liquid nitrogen. The frozen powder was ground and 900 μL of additional TRIzol was added. The frozen lysate was then repeatedly (x10) thawed at 37°C on a heat block, and vortexed for 30 seconds. Then, 200 μl of chloroform was added per 1 ml TRIzol. The sample was mixed by inversion for 15 seconds before incubation at room temperature for 3 minutes for phase separation. The sample was then spun at 12,000 g for 15 mins at 4°C. The upper aqueous phase was then transferred to a fresh tube. We next added an equal volume of 100% ethanol, mixed by pipetting, and transferred the whole to a Qiagen RNeasy spin column, which was processed according to the manufacturer’s instructions. The resulting RNA was measured with the Qubit and the samples were combined to equal concentrations. The RNA was sent to the Center for Genomic and Computational Biology at Duke University for PacBio Sequel Sequencing.

We generated *de novo* gene models generally following the protocol of Doyle et al. (2020). RNAseq reads were mapped to the genome using STAR (Dobin & Gingeras, 2015). The resulting BAM was used as hints for BRAKER2 (Brůna et al., 2021), which trains AUGUSTUS (Stanke et al., 2006) using *ab initio* predictions generated by GeneMark-ES (Ter-Hovhannisyan et al., 2008). Attempts to use mapped Iso-Seq or proteins of closely related Nematodes as input for BRAKER2 yielded poor results. Therefore, only short read RNAseq was used.

To better utilize the Iso-Seq data we mapped it to the genome using PASA (Haas et al., 2003). We then loaded the BRAKER2 annotation into PASA to iteratively update existing annotations based on alignment evidence. We performed two rounds of updates to merge the two annotations following the recommendations of the PASA authors. We incorporated further sources of evidence by mapping *C. elegans* proteins to the *C. becei* genome using Exonerate (Slater & Birney, 2005) with an alignment score threshold of 50%. GFFs were subsequently extracted from the exonerate output with Exonerate_to_evm_gff3.pl from EvidenceModeler (Haas et al., 2008). BRAKER2, PASA, and exonerate annotations were combined using weights of 2, 5, and 2 respectively in EvidenceModeler. The evidence modeler annotations were updated one last time for two rounds in PASA to incorporate additional evidence from long-read transcripts adding any UTR and isoform information that may have been missed or discarded.

### Genomic GC Content

We calculated the GC content with bedtools *nuc* (Quinlan & Hall, 2010). For analyses by functional class, we used species-specific GFF annotation files and computed base composition for each genomic feature. A nested masking approach was employed, where genomes were first masked for exons and subsequently for repeats, allowing for the separation of intergenic and intronic regions from exonic and repetitive sequences. At each masking step, feature-specific GC content was calculated. To assess localized variations, GC content was then calculated in 10 kb windows across the genome for each feature.

### Codon and Amino Acid Usage

To identify differences in codon preference among *Caenorhabditis* species, we analyzed the mean Relative Synonymous Codon Usage (RSCU) values (Sharp & Li, 1986). RSCU is a measure of codon bias, indicating how frequently a given codon is used relative to the expected frequency if all synonymous codons for an amino acid were used equally. An RSCU value of 1 suggests that a codon is used at its expected frequency under no bias, whereas values greater than 1 indicate codon preference, and values less than 1 indicate underrepresentation of that codon. Coding sequences for each species were retrieved from FASTA files, and RSCU values were calculated using the *uco* function from the *seqinr* package in R (Charif & Lobry, 2007).

Amino acid composition was calculated by mapping codons to their corresponding amino acids and summing codon counts for each amino acid across all coding sequences (CDS) in a species.

### Synteny Analysis

To visualize conserved gene order, we identified single-copy orthologs shared among *C. becei*, *C. niphades*, and *C. elegans* and then generated plots connecting the chromosomal positions of these orthologs across species. We found that chromosomes of *C. niphades* (Sun et al., 2022) were syntenic with their corresponding chromosomes in *C. elegans* and *C. becei*, but the order of the genes along five of the chromosomes (I, II, III, V, and X) was largely inverted, suggesting that the genome fasta for these chromosomes represents the reverse complement relative to the other species. Chromosome IV, in contrast, was highly collinear between *C. becei* and *C. niphades*. For the plots in Figure 8, we reversed the orientation of the five *C. niphades* chromosomes to maximize the collinearity.

### Ortholog Identification & Functional Annotation

Orthologous gene families were identified using OrthoFinder2 (Emms & Kelly, 2019) with default parameters. The longest coding DNA sequence for each gene across multiple *Caenorhabditis* species was extracted and used as input. Genes were assigned to orthogroups, representing clusters of orthologous and paralogous genes shared among species. This enabled comparison of gene family expansions, contractions, and species-specific duplications.

To assess chromosomal distributions, the positions of single-copy and duplicated orthologs were mapped using the *C. becei* genome annotation. Gene counts were binned into 100 kb windows, and the distribution of multi-copy, single-copy, and unassigned genes was analyzed across chromosomes. To characterize orthogroups, we calculated gene count and additive length, where additive length represents the sum of all gene lengths within an orthogroup. This approach was used to compare the overall genomic contribution of orthogroups with different copy numbers and to examine patterns of gene family expansion.

Functional annotation of coding sequences was performed using InterProScan v5.65 (Blum et al., 2021), with parameters for nucleotide input, translation, domain prediction, Gene Ontology (GO) assignment, and pathway annotation (interproscan.sh -t n -dp -goterms -pa). InterProScan results were parsed into a non-redundant gene-level table containing the InterPro accession, type, and description, along with associated GO terms and functional descriptions. These annotations were then added to the corresponding gene features in the genome annotation (GFF) file.

## Supplementary Material

Supplement 1 SUPPLEMENTARY FILES
**File S1. G4BC2Simulations**. This R script generates samples of chromosomes under the experimental design used to build the genetic map, under the assumption of complete crossover interference. It then tests the fit of the observed crossover number to the simulation results.
**File S2. Reporter plasmid sequences**. Fasta formatted DNA sequences for pSAS02 and pSAS06, plasmids for expression of male-specific GFP and female specific mCherry.
SUPPLEMENTARY TABLES
**Table S1:** *C. becei* nuclear genome assembly statistics.
**Table S2.** The genetic map of *C. becei*, with physical positions of markers on the genome assembly.
**Table S3.** Domains in *C. becei* and other species From genetic and physical maps for each chromosome, after excluding the terminal megabase from each end, we used segmented linear regression to identify three recombination-rate domains. The table records the positions of the boundaries (LC and CR, boundaries between the left arm and center and between the center and right arm, respectively), the percent of chromosome length that is in the left arm and tip, center domain, and right arm and tip, and then the recombination rates estimated for each domain from the regression slopes. Note that four of the 36 chromosome maps (6 chromosomes × 6 species) have recombination rate patterns that do not match the expectations of the regression, and the numbers in the table for these are not meaningful. These chromosomes – *C. tropicalis* X, *C. remanei* IV and X, and *P. pacificus* X – are indicated by “No” in the “Domains” column in the table. The *P. pacificus* X is likely affected by segregating inversions and so may not reflect the meiotic map in structural homozygotes.
**Table S4.** Gene Count and Additive Length Table for top Orthogroups in chromosome X. This table provides the number of genes and the additive length per orthogroup for *C. becei*. The table includes the orthogroup ID and a brief functional description (if available).
**Table S5.** Transcriptome data sources for the phylogenetic analysis in Figure 1.
**Table S6.** *Medea* data. Results of experimental crosses testing for Medea or Peel activity. These are the data underlying Figure 4B. Each row represents one worm’s progeny, indexed in column 1 to its randomly assigned plate number. Columns 2-10 count the numbers of embryos observed after egg lay, unhatched embryos and deformed larvae the next day, and then wild-type adult females, wild-type adult males, L4 females, L4 males, and L1-3 larvae on the subsequent day. Columns 11 and 12 record the genotypes of the parents of the cross, and column 13 records an identifier for which of the 16 classes of cross the row represents.

Supplement 2

## Data Availability

Newly generated transcriptomes used to assemble the phylogeny in Figure 1 are associated with BioProject ID PRJNA1128046. QG2082 HiFi, HiC, Illumina RNAseq, and PacBioIsoseq data used to assemble and annotate the *C. becei* genome, and the assembly and annotation files, are associated with BioProject PRJNA989223.
