# Supplementary figures and images for: Conservative evolution of genetic and genomic features in Caenorhabditis becei, an experimentally tractable gonochoristic worm
Source: bioRxiv. 2025 May 15:2025.05.09.653148. Preprint. [Version 1] doi: 10.1101/2025.05.09.653148 (PMC12132450; doi:10.1101/2025.05.09.653148)

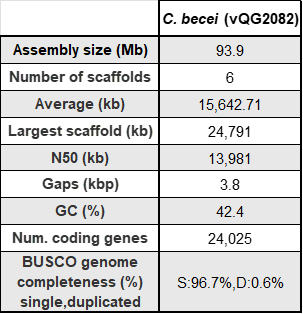

Supplement: Supplement 1 — SUPPLEMENTARY FILES File S1. G4BC2Simulations. This R script generates samples of chromosomes under the experimental design used to build the genetic map, under the assumption of complete crossover interference. It then tests the fit of the observed crossover number to the simulation results. File S2. Reporter plasmid sequences. Fasta formatted DNA sequences for pSAS02 and pSAS06, plasmids for expression of male-specific GFP and female specific mCherry. SUPPLEMENTARY TABLES Table S1: C. becei nuclear genome assembly statistics. Table S2. The genetic map of C. becei, with physical positions of markers on the genome assembly. Table S3. Domains in C. becei and other species From genetic and physical maps for each chromosome, after excluding the terminal megabase from each end, we used segmented linear regression to identify three recombination-rate domains. The table records the positions of the boundaries (LC and CR, boundaries between the left arm and center and between the center and right arm, respectively), the percent of chromosome length that is in the left arm and tip, center domain, and right arm and tip, and then the recombination rates estimated for each domain from the regression slopes. Note that four of the 36 chromosome maps (6 chromosomes × 6 species) have recombination rate patterns that do not match the expectations of the regression, and the numbers in the table for these are not meaningful. These chromosomes – C. tropicalis X, C. remanei IV and X, and P. pacificus X – are indicated by “No” in the “Domains” column in the table. The P. pacificus X is likely affected by segregating inversions and so may not reflect the meiotic map in structural homozygotes. Table S4. Gene Count and Additive Length Table for top Orthogroups in chromosome X. This table provides the number of genes and the additive length per orthogroup for C. becei. The table includes the orthogroup ID and a brief functional description (if available). Table S5. Transcriptome data source [file media-1.zip › SupplementaryFiles/TableS1.GenomeStats.png]
