# Supplementary material for: Conservative evolution of genetic and genomic features in Caenorhabditis becei, an experimentally tractable gonochoristic worm
Source: bioRxiv. 2025 May 15:2025.05.09.653148. Preprint. [Version 1] doi: 10.1101/2025.05.09.653148 (PMC12132450; doi:10.1101/2025.05.09.653148)
Supplement: Supplement 2 [file NIHPP2025.05.09.653148v1-supplement-2.pdf]

bioRxiv preprint doi: https://doi.org/10.1101/2025.05.09.653148; this version posted May 15, 2025. The copyright holder for this preprint (which was not certified by peer review) is the author/funder, who has granted bioRxiv a license to display the preprint in perpetuity. It is made available under aCC-BY 4.0 International license.

Conservative evolution of genetic and genomic features in *Caenorhabditis becei*, an experimentally tractable gonochoristic worm

SUPPLEMENTARY FIGURES

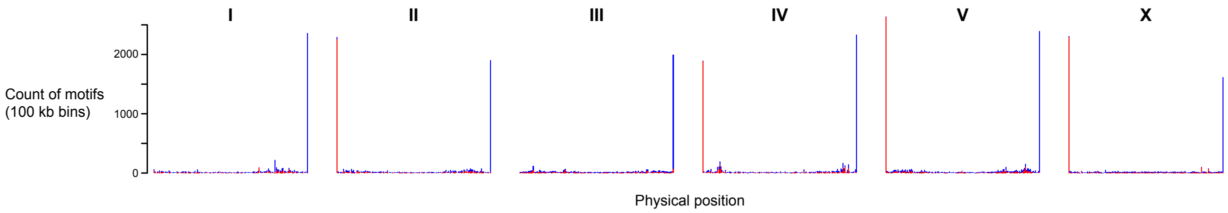

Figure S1. Chromosomes end with oriented telomere sequences in most cases. The plot shows stacked histograms of the counts of TTAGGC (blue) and GCCTAA (red), in 100kb bins along each chromosome. The left ends of chromosomes I and III lack telomere sequences.

bioRxiv preprint doi: https://doi.org/10.1101/2025.05.09.653148; this version posted May 15, 2025. The copyright holder for this preprint (which was not certified by peer review) is the author/funder, who has granted bioRxiv a license to display the preprint in perpetuity. It is made available under aCC-BY 4.0 International license.

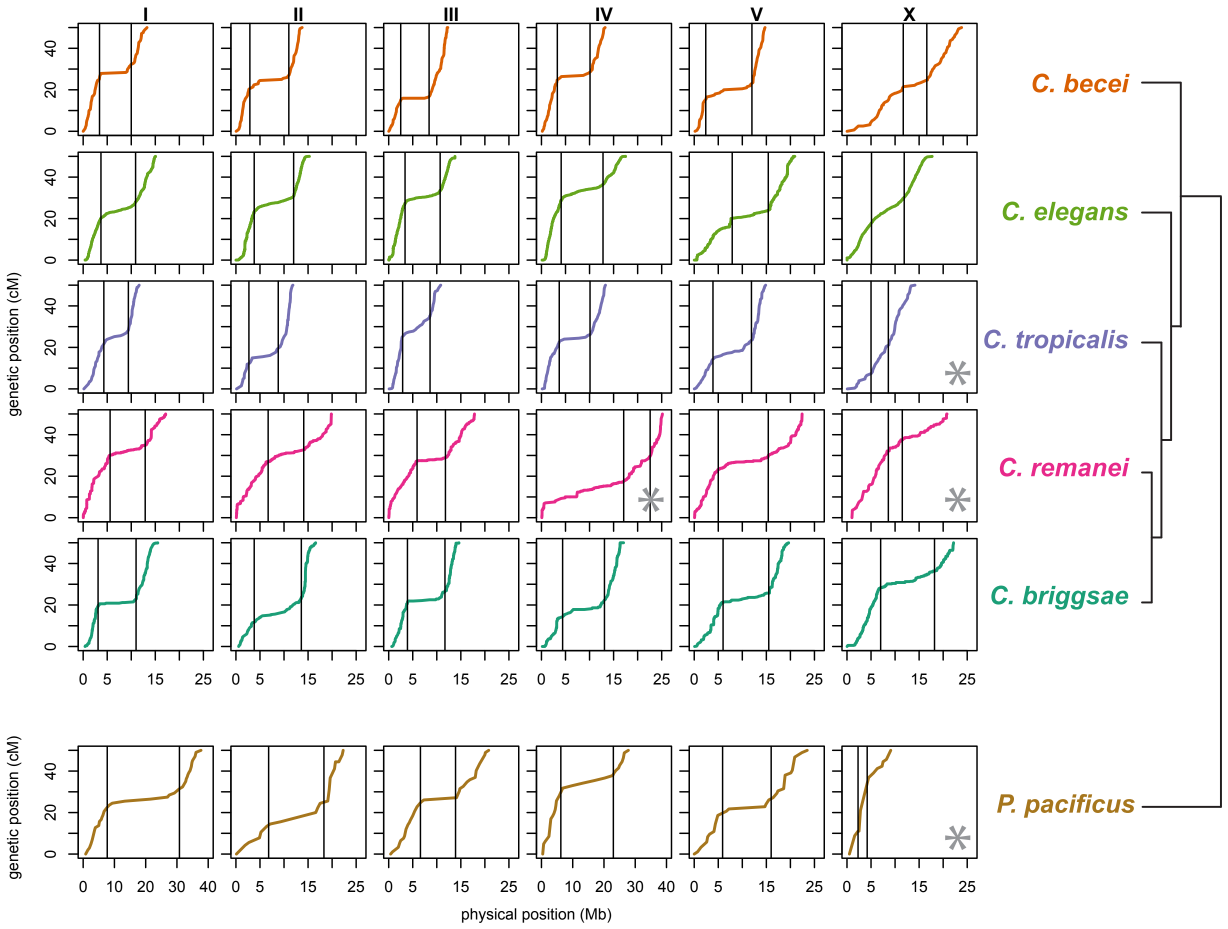

Figure S2. Domains in *C. becei* and other species. Marey maps for each of the chromosomes in six species with genetic map data. Vertical lines mark the estimated positions of chromosome domain boundaries. The x-axis is the physical position along the chromosome, in Mb, and the y-axis is the genetic position, in cM, after rescaling each map to 50 cM total length. The x-axis runs from 0 to 26 Mb in each plot, with the exceptions of *P. pacificus* chromosomes I and IV, which are much longer. As described in Table S2, four chromosome maps (marked with asterisks) do not have the expected domain structure: *C. tropicalis* X, *C. remanei* IV and X, and *P. pacificus* X.

bioRxiv preprint doi: https://doi.org/10.1101/2025.05.09.653148; this version posted May 15, 2025. The copyright holder for this preprint (which was not certified by peer review) is the author/funder, who has granted bioRxiv a license to display the preprint in perpetuity. It is made available under aCC-BY 4.0 International license.

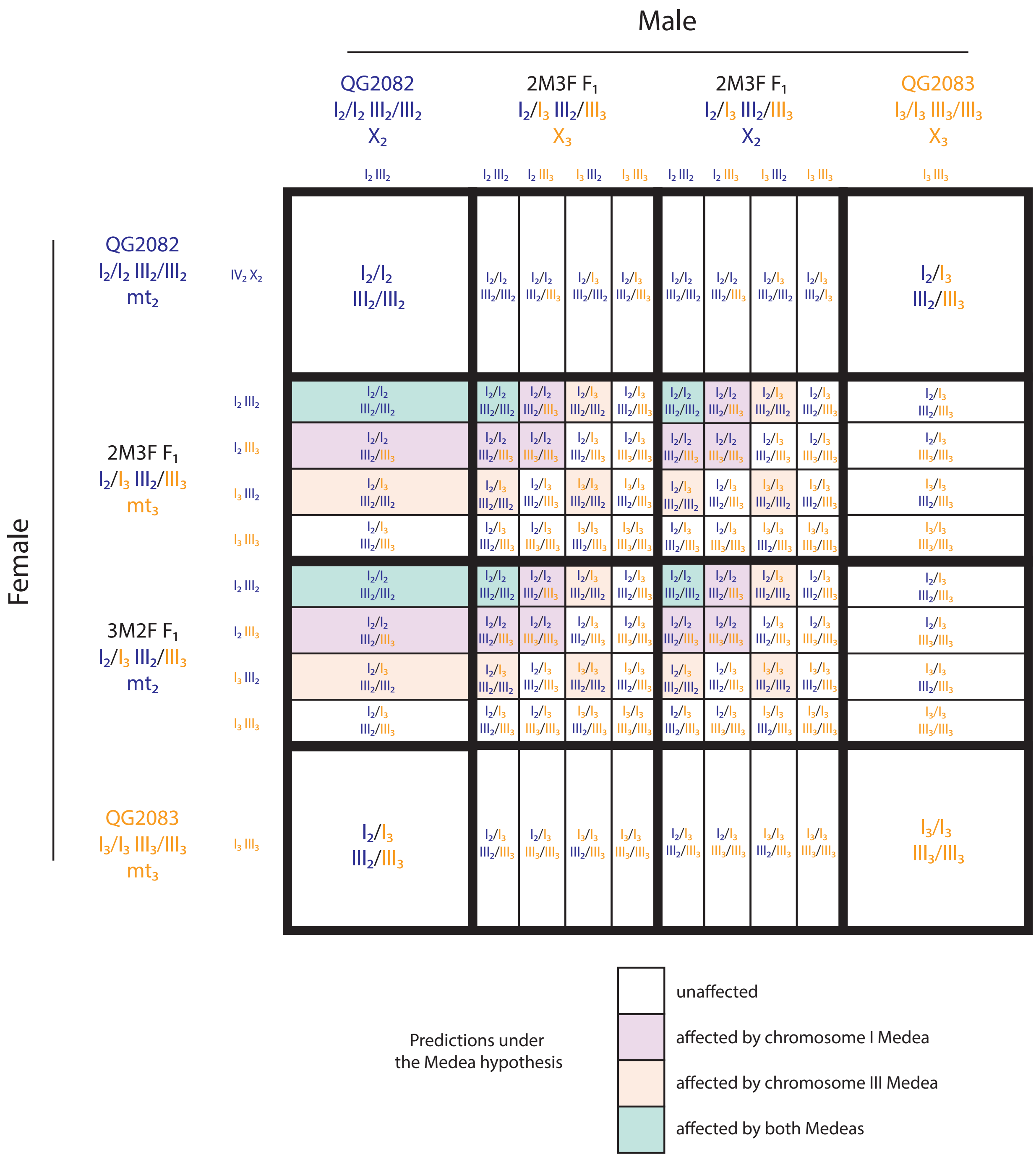

Figure S3. Sixteen Punnett squares showing the expected frequencies of affected progeny under a model of *Medea* elements on chromosomes I and III in QG2083 that independently affect QG2082-homozygous progeny of heterozygous mothers.

bioRxiv preprint doi: https://doi.org/10.1101/2025.05.09.653148; this version posted May 15, 2025. The copyright holder for this preprint (which was not certified by peer review) is the author/funder, who has granted bioRxiv a license to display the preprint in perpetuity. It is made available under aCC-BY 4.0 International license.

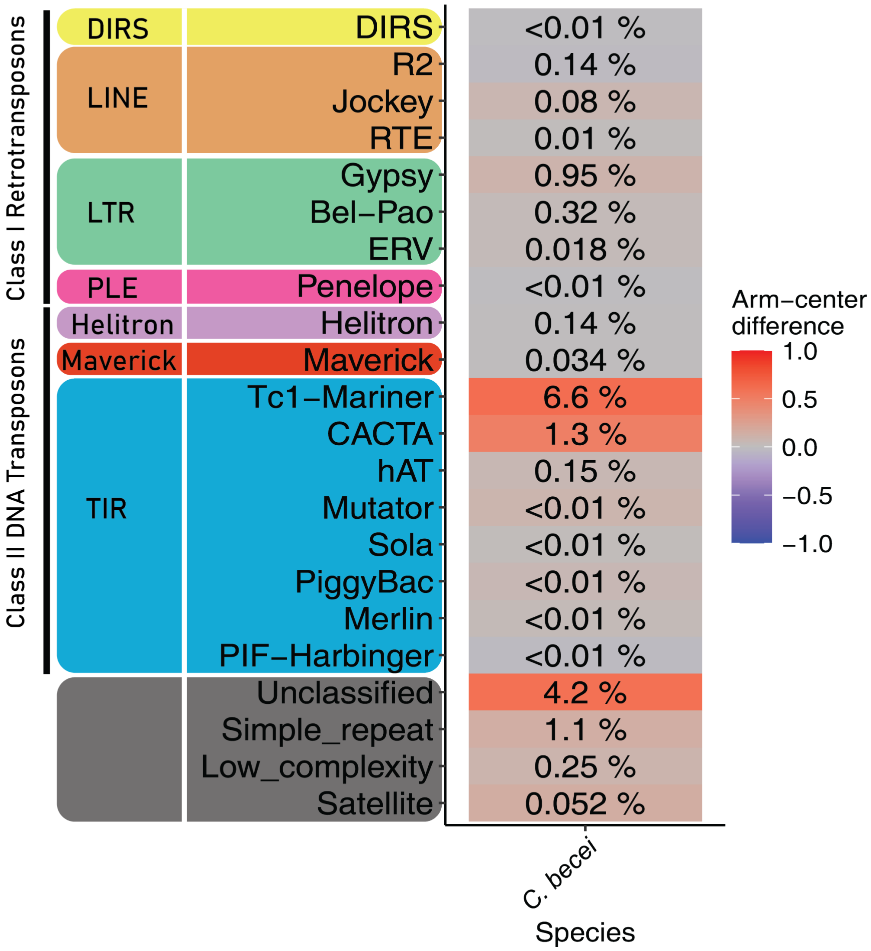

Figure S4. Transposable element superfamilies in *C. becei*. Each row represents a transposable element (TE) superfamily, grouped by higher-level taxonomic categories and colored by repeat order. Colored boxes next to each superfamily indicate the arm-center difference (Cohen's d), calculated as the difference in mean repeat density between chromosome arms (normalized position ≥ 0.25) and centers (normalized position < 0.25), divided by the pooled standard deviation. Positive values (red) reflect higher repeat density in chromosome arms, negative values (blue) indicate enrichment in chromosome centers, and values near zero are shown in grey. Numbers within boxes show the percentage of the genome occupied by each superfamily.

bioRxiv preprint doi: https://doi.org/10.1101/2025.05.09.653148; this version posted May 15, 2025. The copyright holder for this preprint (which was not certified by peer review) is the author/funder, who has granted bioRxiv a license to display the preprint in perpetuity. It is made available under aCC-BY 4.0 International license.

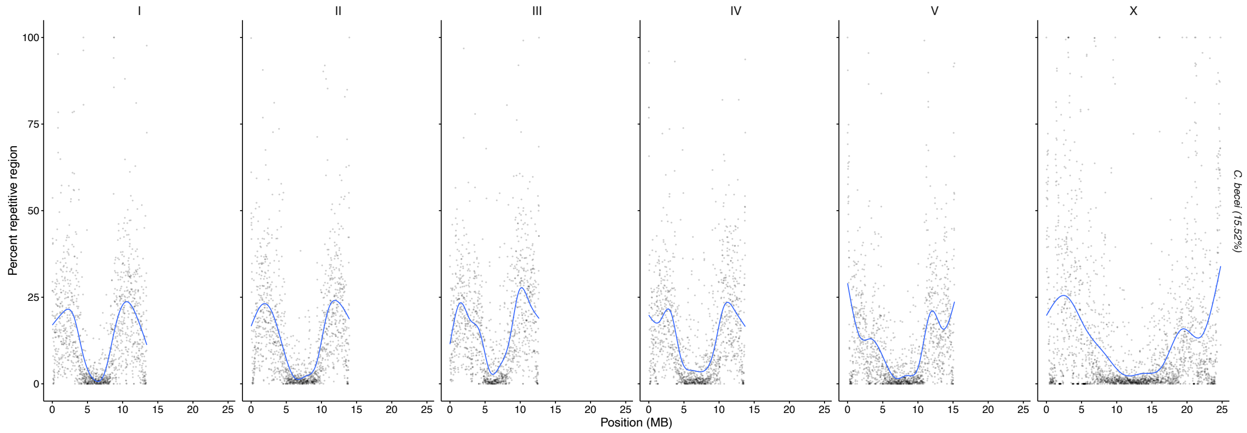

Figure S5. Global repetitive element landscape across 10-kb windows along the length of the chromosomes in *C. becei*. Blue lines show the smoothed trend in repeat density, generated by fitting a generalized additive model to the data.

bioRxiv preprint doi: https://doi.org/10.1101/2025.05.09.653148; this version posted May 15, 2025. The copyright holder for this preprint (which was not certified by peer review) is the author/funder, who has granted bioRxiv a license to display the preprint in perpetuity. It is made available under aCC-BY 4.0 International license.

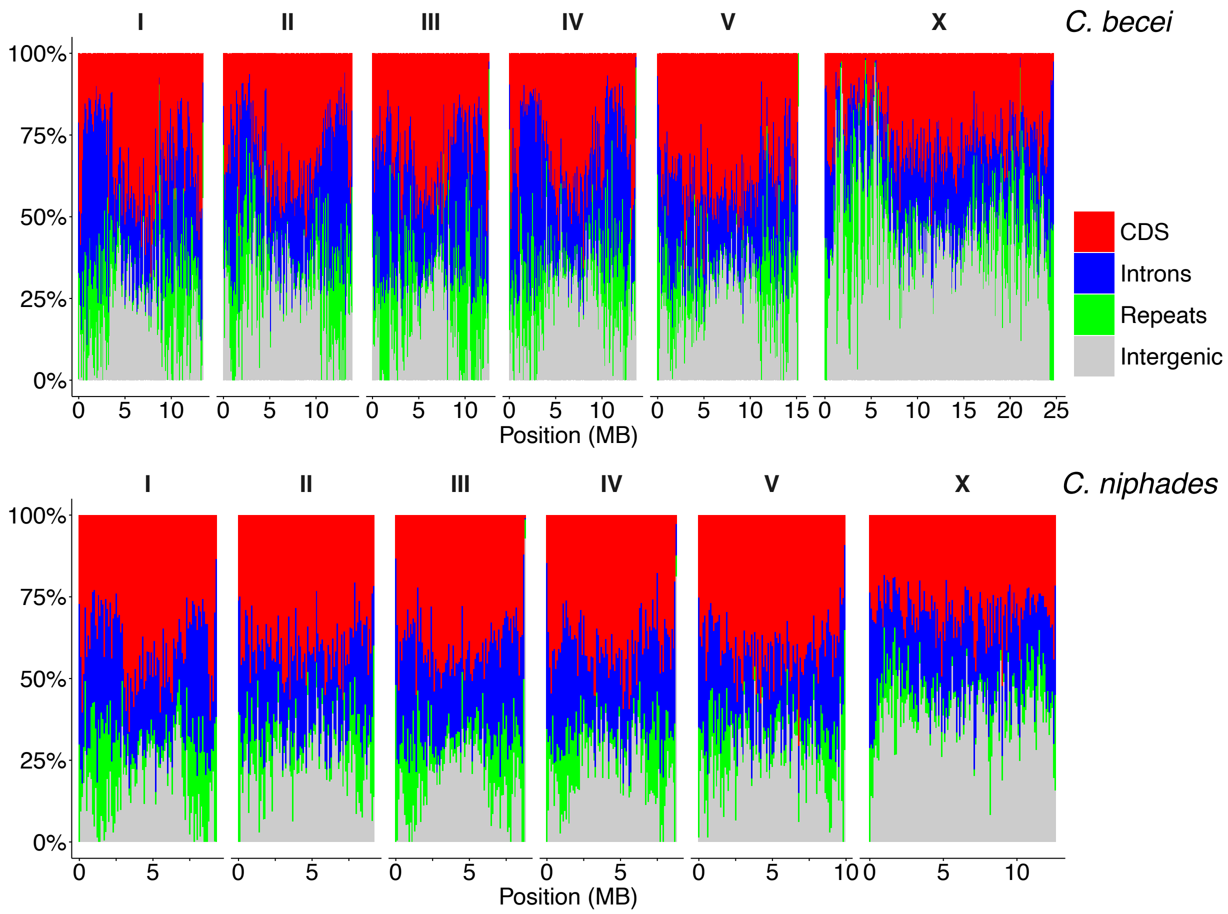

Figure S6. Genomic feature composition across the chromosomes of *C. becei* and *C. niphades***.** The proportion of each 100-kb window occupied by coding (CDS, red), intronic (blue), repetitive (green), and intergenic (gray) bases is shown along each chromosome. Bars are stacked to sum to 100% per window. Intergenic regions represent sequence not annotated as CDS, intron, or repeat.

bioRxiv preprint doi: https://doi.org/10.1101/2025.05.09.653148; this version posted May 15, 2025. The copyright holder for this preprint (which was not certified by peer review) is the author/funder, who has granted bioRxiv a license to display the preprint in perpetuity. It is made available under aCC-BY 4.0 International license.

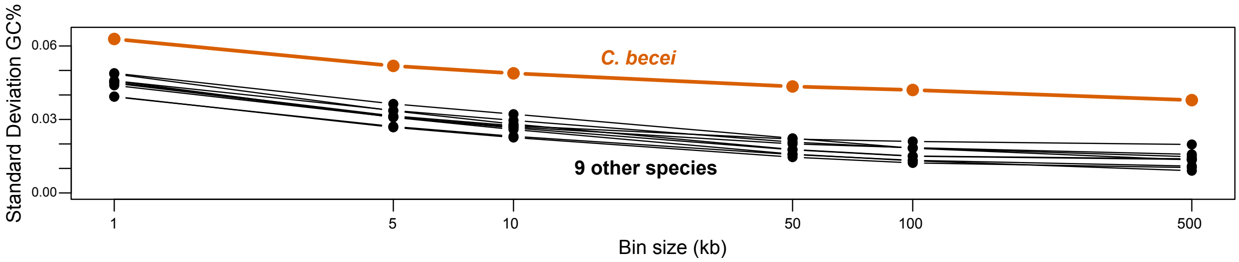

Figure S7. The *C. becei* genome is unusually variable in its local GC percentage. Here the standard deviation of GC% across the genome is shown as a function of the length scale over which GC is measured, in bins of 1 to 500 kb. The spacing along the x-axis is logged. The nine species shown in comparison to *C. becei* are those plotted in Figure 5.

bioRxiv preprint doi: https://doi.org/10.1101/2025.05.09.653148; this version posted May 15, 2025. The copyright holder for this preprint (which was not certified by peer review) is the author/funder, who has granted bioRxiv a license to display the preprint in perpetuity. It is made available under aCC-BY 4.0 International license.

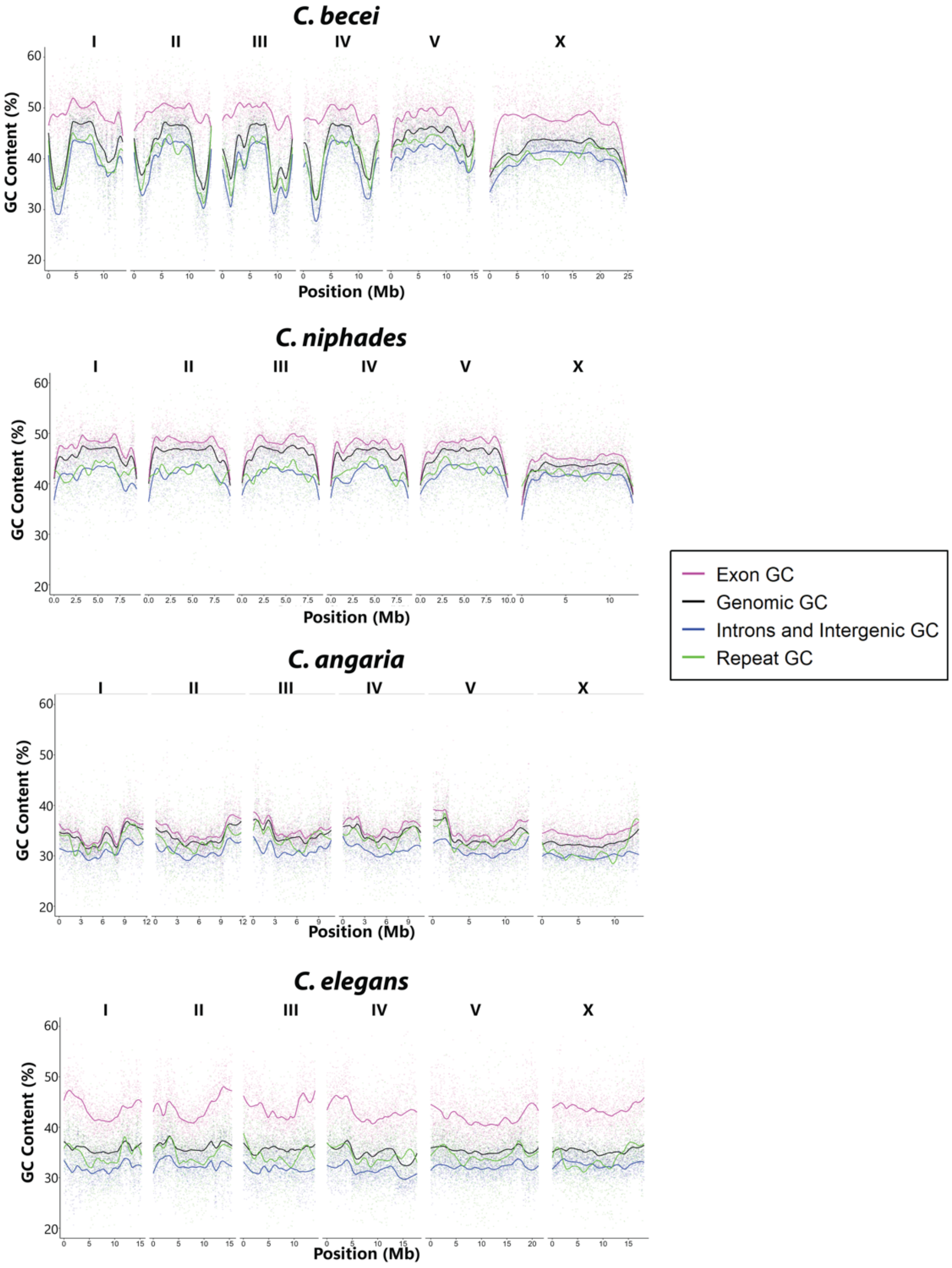

Figure S8. GC content of genomic features in *Caenorhabditis* species. GC content was calculated from the counts of G+C divided by the total number of bases of the feature within non-overlapping 10 kb windows along the length of the chromosome, with LOESS-fitted lines (span = 0.2). *C. elegans* here represents the relatively homogenous Elegans Group species.

bioRxiv preprint doi: https://doi.org/10.1101/2025.05.09.653148; this version posted May 15, 2025. The copyright holder for this preprint (which was not certified by peer review) is the author/funder, who has granted bioRxiv a license to display the preprint in perpetuity. It is made available under aCC-BY 4.0 International license.

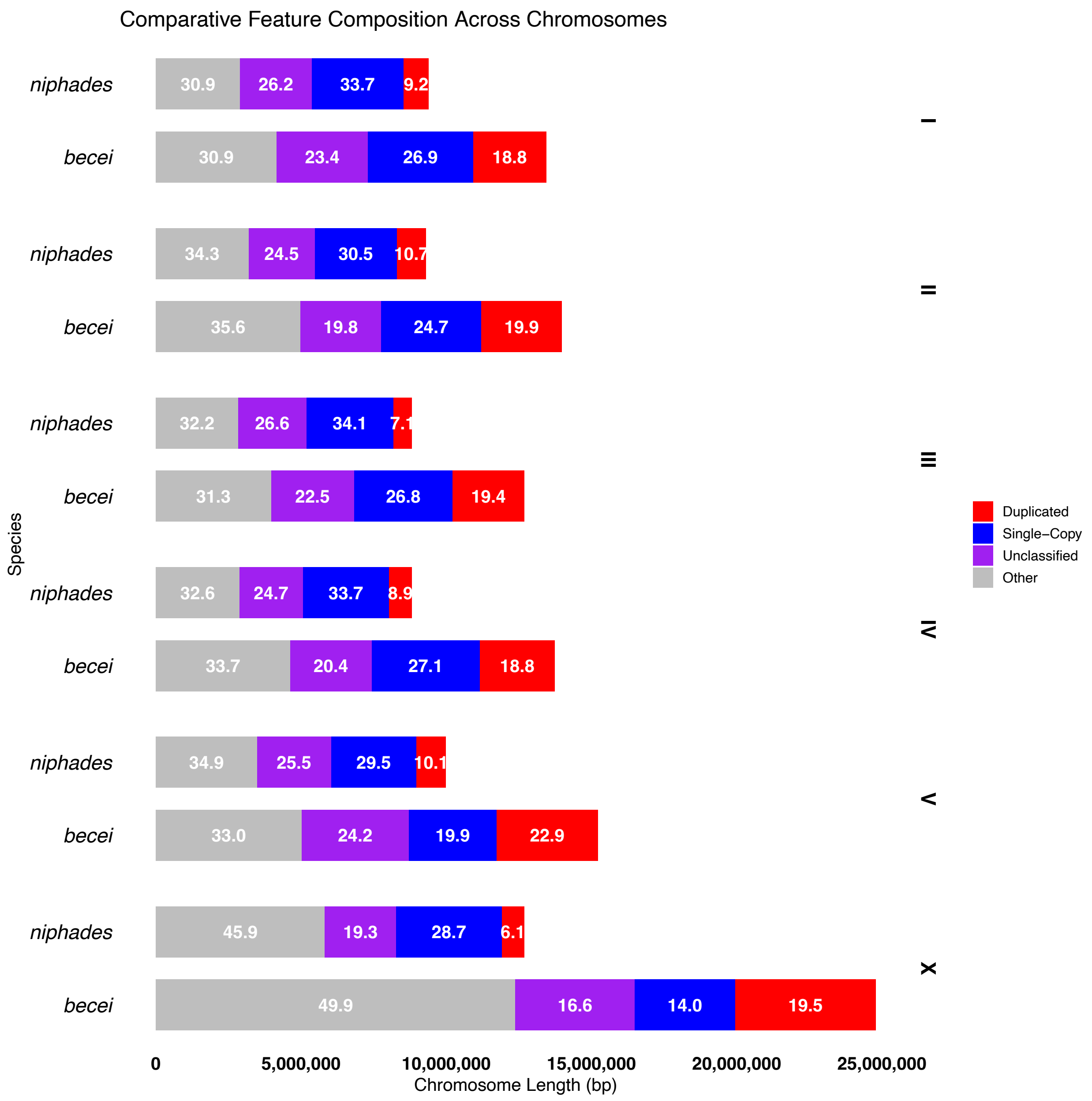

Figure S9. Chromosome size and ortholog classification of CDS and introns in *C. becei* and *C. niphades*. The total length of gene regions (CDS + introns) and intergenic DNA is shown for each chromosome. Gene regions were merged and classified based on orthogroup analysis into four categories: duplicated (red), single-copy (blue), unclassified (purple), and intergenic (gray). Percentages within bars indicate the proportion of each feature relative to chromosome size.

bioRxiv preprint doi: https://doi.org/10.1101/2025.05.09.653148; this version posted May 15, 2025. The copyright holder for this preprint (which was not certified by peer review) is the author/funder, who has granted bioRxiv a license to display the preprint in perpetuity. It is made available under aCC-BY 4.0 International license.

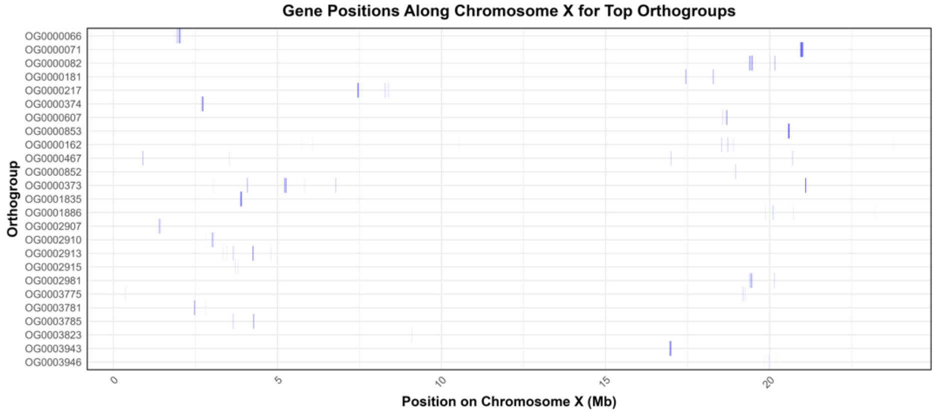

Orthogroup	Gene_Count	Additive_Length
OG0000066	30	33426
OG0000071	28	52651
OG0000082	23	41089
OG0000181	18	19324
OG0000217	17	27573
OG0000374	13	24415
OG0000607	12	15385
OG0000853	11	27913
OG0000162	10	13618
OG0000467	10	17232
OG0000852	10	4606
OG0000373	9	53088
OG0001835	8	24946
OG0001886	8	9011
OG0002907	8	12824
OG0002910	8	12887
OG0002913	8	22381
OG0002915	8	4072
OG0002981	8	22725
OG0003775	7	8362
OG0003781	7	11555
OG0003785	7	14808
OG0003823	7	2046
OG0003943	7	24673
OG0003946	7	3925

Figure S10. Gene positions along Chromosome X for top orthogroups by count. The figure displays the positions of genes from the orthogroups with highest number of genes in chromosome X of *C. becei*. Each horizontal blue line represents the start and end positions of genes for a given orthogroup. The x-axis indicates the chromosomal position in megabases (Mb), while the y-axis shows the orthogroup identifiers, sorted by gene count.
